# Structural insights and rational design of *Pseudomonasputida* KT2440 omega transaminases for enhanced biotransformation of (*R*)-PAC to (1*R*, 2*S*)-Norephedrine

**DOI:** 10.1016/j.jbc.2025.110289

**Published:** 2025-05-26

**Authors:** Parijat Das, Santosh Noronha, Prasenjit Bhaumik

**Affiliations:** 1 Department of Biosciences and Bioengineering, Indian Institute of Technology Bombay, Mumbai, India; 2 Department of Chemical Engineering, Indian Institute of Technology Bombay, Mumbai, India

**Keywords:** omega transaminases, rational design, biotransformation, X-ray crystallography, structural biology, enzyme structure, enzyme catalysis, structure–function

## Abstract

Omega transaminases (ω-TAs) can mediate the chiral amination of several unnatural substrates without the requirement of an α-COOH group and are highly relevant in the production of several pharmaceutical intermediates of commercial interest. Development of better variants of ω-TAs is hence essential for the biotransformation of unnatural substrates. We studied the active site architecture of the wild-type ω-TAs, to engineer enzymes that enhance the biotransformation of (*R*)-phenylacetylcarbinol to (1*R*, 2*S*)-norephedrine. Two such ω-TAs (TA_5182 and TA_2799) from *P. putida* KT2440 strain were overexpressed and purified as recombinant proteins. Crystal structures of TA_5182 were solved in two conformations, revealing significant movements of two highly flexible loops in these different states. The TA_2799 structure was determined as a complex with the cofactor pyridoxal 5ʹ-phosphate (PLP) covalently bound to the catalytic K286 as an internal aldimine. Enzyme assays indicated that TA_2799 required a four-fold higher cofactor concentration than TA_5182 to achieve satisfactory biotransformation of (*R*)-PAC. A key mutation of L322F in TA_2799 drastically reduced (∼8-fold) the cofactor dependency of the TA_2799_L322F mutant enzyme, and the mutant remained active for 96 h at 30 °C. The crystal structure of the mutant enzyme revealed a key asparagine residue that mediates a hydrogen bonding network at the dimeric interface of the enzyme and is absent in TA_5182. The TA_5182_G119N mutant also showed enhanced cofactor affinity. The results of our studies will help generate *Pseudomonad* ω-TAs and ω-TAs from other organisms with high efficiency for asymmetric synthesis, for further applications in large-scale biotransformation processes.

Transaminases (TAs) or aminotransferases are a class of enzymes that catalyze the exchange of an amine (-NH_2_) group with a keto (C=O) group between an amino acid and an α-keto acid. These enzymes are highly stereoselective and have two substrate-binding pockets of varying sizes along with the cofactor pyridoxal 5ʹ-phosphate (PLP). During transamination, the binding and involvement of the PLP molecule to form a Schiff base intermediate is one of the key steps in catalysis (1). While α- and β-TAs require a -COOH moiety for catalysis, ω-TAs can catalyze the transamination reaction without the requirement of the -COOH moiety. Therefore, ω-TAs can accept a wide range of donor and acceptor substrates, leading to their potential use to synthesize commercially important compounds (2). Due to their high enantiomeric selectivity and environmentally friendly, concise reactions, transaminases have broad industrial applications, especially in the pharmaceutical industry (3).

In recent years, biocatalytic routes have emerged as promising approaches for producing various bulk and fine chemicals, including pharmaceuticals (4). Among the significant advancements in this area, the biocatalytic synthesis of chiral amines has garnered considerable attention. Chiral amines, with their diverse applications as drugs and chiral auxiliaries in organic synthesis, play a crucial role in the pharmaceutical and chemical industries. One such industrially important chiral amine is (1*R,* 2*S*)-norephedrine ((1*R,* 2*S*)-NE). (1*R*, 2*S*)-NE and its derivatives primarily serve as chiral auxiliaries or chiral catalysts in various asymmetric reactions (5, 6). Chemical synthesis of (1*R*, 2*S*)-NE from (*R*)-phenylacetylcarbinol ((*R*)-PAC) poses several challenges, including low enantioselectivity and the requirement for multiple synthesis steps involving harsh reaction conditions (5, 7). As a result, researchers have adopted biocatalytic strategies, utilizing transaminases as stereoselective biocatalysts for chiral amine synthesis (6, 8, 9) (Fig. 1). Despite their industrial importance, most reported transaminases exhibit insufficient operational or kinetic stability at high substrate concentrations, in denaturing solvents, and at elevated temperatures (4, 10). Thus, there is a need for the development of better variants of transaminases for industrial use. Research has focused on understanding the molecular basis of the kinetic instability of transaminases for their rational engineering. Studies on the well-characterized transaminase from *Chromobacterium violaceum* (*Cv-*TA) indicate that PLP binding, especially the binding of the 5ʹ-phosphate in the phosphate group binding cup (PGBC), acts as the driving factor for the structural rearrangements in the enzyme (11, 12). In this context, the affinity of transaminases toward the cofactor PLP plays a key role in maintaining the stability of the enzyme (10). In bacterial fold type I ω-TAs, two monomers are required to form the active site and the PLP binding pocket. The binding of the cofactor depends on the dynamics of the two flexible loops. The N-terminal loop acts as a “lid” covering the aromatic moiety of the PLP molecule, and it interacts with the C-terminal loop (termed as the “roof”) from the other monomer to accommodate the cofactor molecule in the active site of the enzyme. Increasing the affinity of the enzyme towards PLP enhances its stability and biophysical properties (10, 13, 14, 15). Over the last decade, studies have focused on (*S*)- selective ω-TAs belonging to fold type I from several organisms, like *C. violaceum* (11)*, V. fluvalis* (16)*, P. putida* (17)*,* and *H. elongata* (18), that highlight some of the key residues that mediate polar interactions with the cofactor molecule and the movement of the loop during transition of the enzyme from its apo to holo form. However, the effect of the loop movement on enzyme activity and cofactor affinity remains unclear.

**Figure 1:**
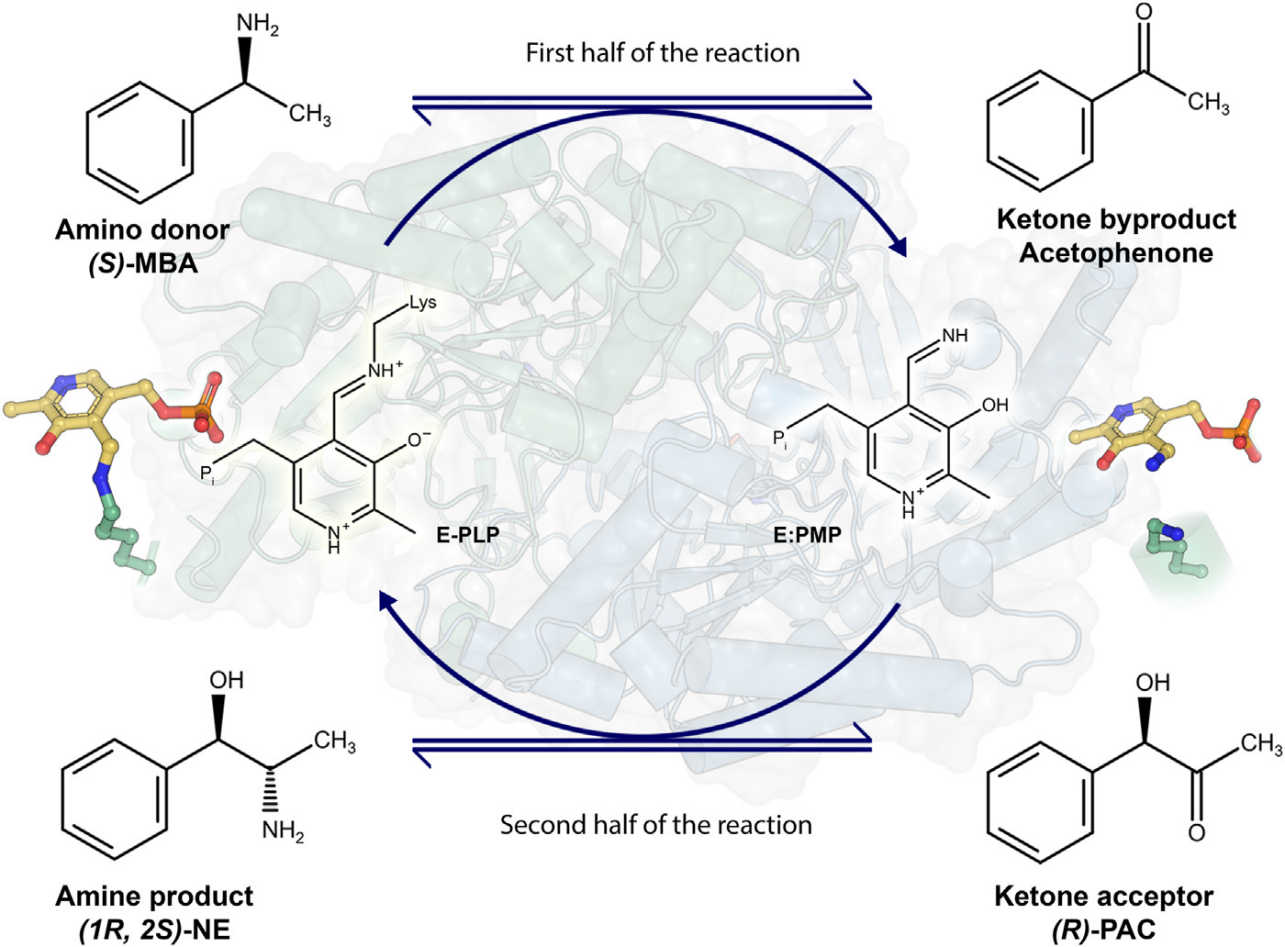
**Reaction pathway of ω-TAs in the formation of (1*R*, 2*S*)*-*Norephedrine ((1*R*, 2*S*)*-*NE).** In the first half of the reaction, the amine group is transferred from the amine donor (*S*)-Methylbenzylamine ((*S*)-MBA) to the PLP molecule to form Pyridoxamine 5′-Phosphate (PMP) which is no longer covalently bound to the catalytic lysine of the enzyme. Acetophenone is produced as a by-product. In the second half of the reaction, the PMP molecule transfers the amine group to the acceptor (*R*)-PAC to form the amine product (1*R*, 2*S*)-NE and the enzyme is regenerated by forming the internal aldimine between PLP and the catalytic lysine.

Transaminases have shown promise in efficiently consuming the substrate (*R*)-PAC for (1*R*, 2*S*)-NE synthesis. Among them, two transaminases TA_2799 (UniProt ID: Q88J50) and TA_5182 (UniProt ID: Q88CJ8) from *Pseudomonas putida* KT2440 have been previously reported for their catalytic potential towards the biocatalysis of organic compounds (17) and formation of (1*R*, 2*S*)-NE (9, 19). However, there is a lack of detailed studies on the enzymatic synthesis of (1*R*, 2*S*)-NE using these specific enzymes. Therefore, to address this gap, comprehensive systematic studies of the transaminases from *P. putida* KT2440 are essential to evaluate their suitability for the enzymatic synthesis of (1*R*, 2*S*)-NE.

In this work, we successfully overexpressed and characterized the two recombinant transaminases TA_2799 and TA_5182 from *P. putida* KT2440 and solved their crystal structures. We determined the structure of TA_5182 in both relaxed (open) and closed states, mimicking the cofactor-free and the cofactor-bound form of the enzyme, respectively. The “roof” loop movement observed in these two forms revealed some important interactions essential for binding of the phosphate group of the cofactor molecule. Furthermore, using a structure-based rational design approach, key residues around the cofactor binding pocket of TA_2799 were mutated, and these single mutants exhibited increased affinity of the enzyme toward PLP. As a result, the mutants showed increased stability and enhanced biophysical properties, making them potential candidates for large-scale biocatalysis of (1*R*, 2*S*)-NE. Our findings are generally applicable to other transaminases and will aid in developing better ω-TAs for industrial applications.

## Results

### Expression and purification of the wild-type TA enzymes and their stability

TA_2799 and TA_5182 were overexpressed in *E. coli* BL21 cells and purified using one-step Ni-NTA affinity chromatography and stored in a buffer containing 50 mM HEPES, 200 mM NaCl at pH 8.0 for biochemical characterization. TA_2799 posed a peculiar problem after the purification of the enzyme. Even under incubation with 50 mM PLP at 4 °C, the enzyme tended to precipitate within 24 h when concentrated over 5 mg/ml. Precipitation was hastened with an increment in temperature and enzyme concentrations. On the other hand, TA_5182 remained stable in solution at a high concentration of 20 mg/ml. Storage instability is commonly observed in wild-type ω-TAs (13), which makes them unsuitable for biocatalytic reactions. We observed a significant drop in enzyme activity (less than 20% activity) within 24 h of purification of the enzyme. To address this problem with TA_2799, we added glycerol to the freshly purified protein as an additive. It was found that a 12.5% (v/v) glycerol mixture effectively kept the enzyme stable in solution (kept in the dark, 4 °C in a freezer), similar to the observation by Chen *et al.* with *C. violaceum* TA (13). This can be associated with the fact that glycerol prevents the aggregation of enzyme molecules and thus prevents precipitation (20). We proceeded to crystallize the enzymes in their apo and PLP-bound states to understand the structural basis of such a contrasting observation.

### Crystal structures of TA_2799 and TA_5182

The crystal structure of TA_2799 was determined at a resolution of 1.76 Å in the PLP-bound holo form. The structure of TA_5182 was solved in two different conformations in its apo state, at resolutions of 3.4 Å and 2.8 Å respectively. The crystal structure of L322F mutant of TA_2799 was solved at a resolution of 2.5 Å. The refinement statistics are summarized in Table 1.

**Table 1:** Data collection and refinement statistics of apo TA_5182 (open and closed forms), PLP bound TA_2799 and apo TA_2799 L322F mutant

	TA_5182 open	TA_5182 closed	TA_2799	TA_2799_L322F
Data collection statistics^a^				
Space group	*P* 2_1_2_1_2_1_	*P* 1	*P* 2_1_2_1_2_1_	*P* 2_1_
Unit cell parameters				
*a*, *b*, *c* (Å)	95.03, 151.34, 156.65	72.57, 72.66, 106.91	75.00, 92.79, 136.25	119.16, 121.44, 147.41
α, β, γ (°)	90.0, 90.0, 90.0	95.67, 107.84, 109.19	90.0, 90.0, 90.0	90.0, 111.88, 90.0
Temperature (K)	100	100	100	100
Wavelength (Å)	1.5418	1.5418	1.5418	0.9789
Resolution (Å)	50.0–3.4 (3.5–3.4)	40.0–2.8 (3.0–2.8)	44.31–1.76 (1.81–1.76)	40.0–2.5 (2.6–2.5)
*R*_merge_ (%)	32.0 (141.3)	10.5 (63.2)	6.6 (24.7)	18.1 (118.5)
Completeness (%)	99.9 (99.9)	93.7 (95.0)	99.2 (98.2)	98.8 (98.5)
Mean *I/σ(I)*	6.16 (1.54)	5.80 (1.36)	18.46 (6.57)	8.71 (1.33)
Unique reflections	31,744 (2594)	44,225 (8374)	94,029 (6834)	133,254 (14,679)
Redundancy	7.35 (7.43)	1.99 (1.99)	6.31 (5.68)	3.89 (3.87)
CC_1/2_ (%)	98.6 (56.6)	98.8 (62.6)	99.8 (96.3)	99.2 (50.6)
Refinement statistics				
Resolution (Å)	48.02–3.40	39.37–2.80	44.31–1.76	39.48–2.50
Working set: number of reflections	30,155	42,013	89,314	126,588
*R*_work_ (%)	20.56	24.74	14.54	19.49
Test set: number of reflections	1588	2212	4702	6663
*R*_free_ (%)	25.80	29.74	17.51	23.28
Protein chains	4	4	2	8
Number of other solvent molecules	13	8	16	26
Number of PLP molecules	--	--	2	--
Number of water molecules	--	45	1012	302
Geometry statistics				
RMSD (bonds) (Å)	0.005	0.004	0.006	0.006
RMSD (angles) (°)	1.339	1.276	0.872	1.289
Ramachandran plot				
Favoured (%)	87.6	92.7	96.7	95.9
Allowed (%)	10.2	5.5	3.1	3.9
Outliers (%)	2.2	1.8	0.2	0.2
PDB ID	9J4Z	9J50	9J2K	9J4Y

a Values in parentheses correspond to the highest resolution shell.

**Figure 2:**
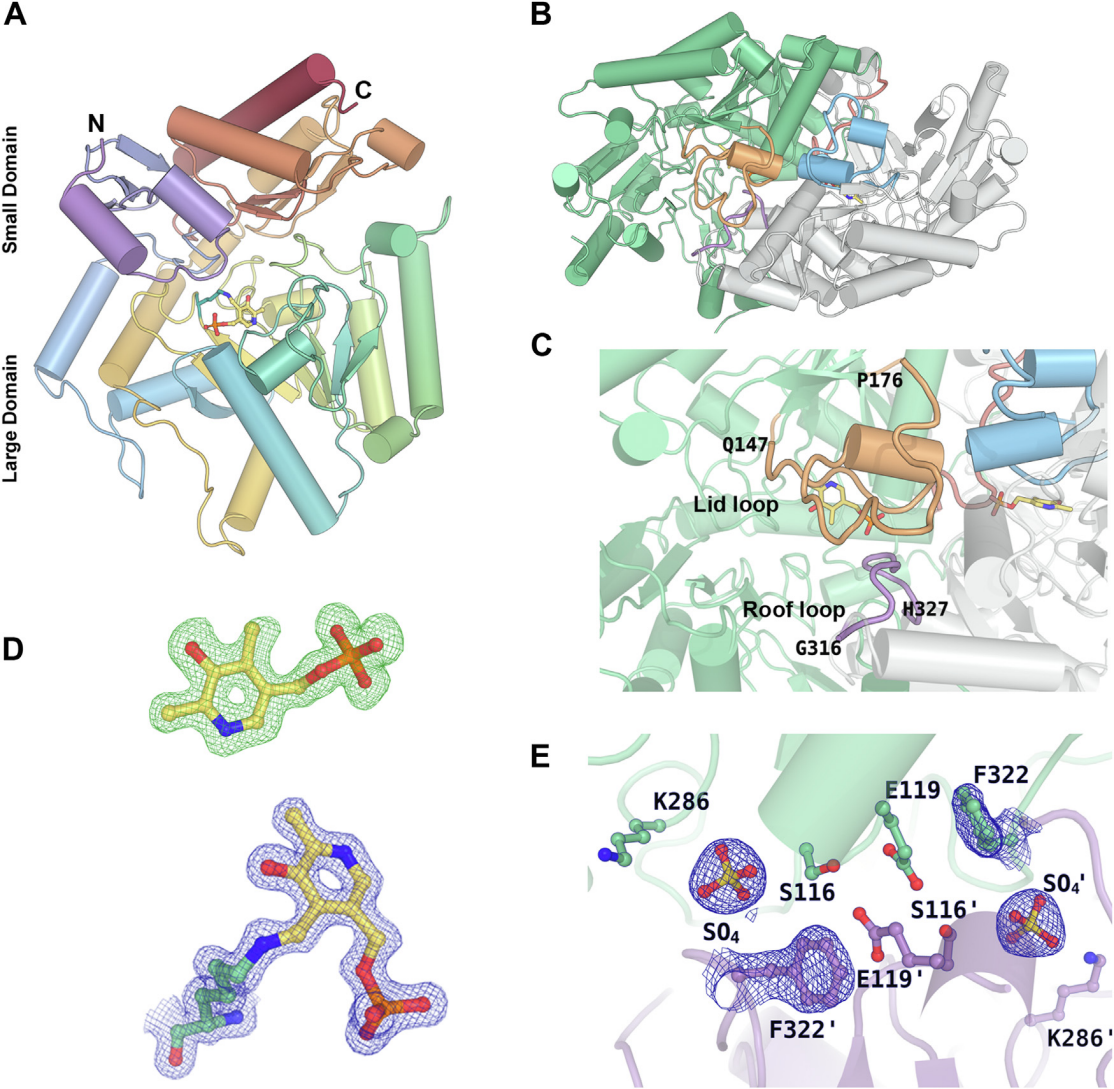
**Overall structure and flexible loops in fold type I ω-TAs.***A*, cartoon representation of the monomer of TA_2799 in rainbow spectrum showing the large and small domains. The PLP molecule covalently bound to the catalytic K286 is represented in *ball* and *stick*. *B*, dimeric view of TA_2799 showing the flexible ‘lid’ and ‘roof’ loops. *C*, zoomed in view of the lid (*orange*) and roof (*purple*) loops. *D*, σ-A weighted *F*_*o*_*-F*_*c*_ omit map (*green mesh*) of PLP contoured at the 3.0 σ level superposed with the final refined model of PLP. Continuous electron density (*blue mesh*) can be observed after fitting the PLP covalently bound to the catalytic K286 in TA_2799 in the *2F*_*o*_*-F*_*c*_ map contoured at 1.0 σ level. *E*, active site architecture of TA_2799_L322F mutant with sulfate ion at the phosphate group binding cup of the enzyme. The *2F*_*o*_*-F*_*c*_ map contoured at 1.0 σ level (*blue mesh*) shows the electron density of the sulfate ion and F322 residue. The prime mark indicates the residues from another subunit of a dimer.

The overall structural fold of the TA_5182 and TA_2799 enzymes is similar to that observed for other class III fold type I TAs with two α/β domains (Fig. 2*A*). The enzymes function as homodimers. For a better understanding of the contributions of the two subunits of the dimer, some of the residues are marked with a prime symbol to indicate the involvement of those residues from another monomer. Each monomer is composed of two major domains, the larger domain that consists of the residues 66 to 344, and the smaller N-terminal domain that consists of the residues 1 to 65 and the C-terminal region of 345 to 459 (Media S1). The large domain has an α/β/α sandwich topology and is centered around a seven-stranded β-sheet as observed in other fold type I ω-TAs (21). The small domain is made up of two β-sheet assemblies. The N-terminal β-sheet assembly is a three-stranded anti-parallel β-sheet instead of being four-stranded as found in some ω-TAs like the *C. violaceum* TA and *Pseudomonas aeruginosa* TAs, where the last β-strand comes from the C-terminal part of the domain (22). This β-sheet assembly is capped by two flexible helices comprising of residues 1 to 32, electron densities of which were observed in the crystal structure of TA_2799 and the closed state of TA_5182. In the crystal structure of the open state of TA_5182, these residues could not be built due to poor electron density in this region and hence were excluded from the final refined model. The C-terminal β-sheet is four-stranded with direction + - + + with a topology of +1, +2×, −1. As seen in other TAs, this sheet is shielded on one side from the solvent by three α-helices. The other side forms a crevice with the large domain, which accommodates the enzyme’s active site.

**Figure 3:**
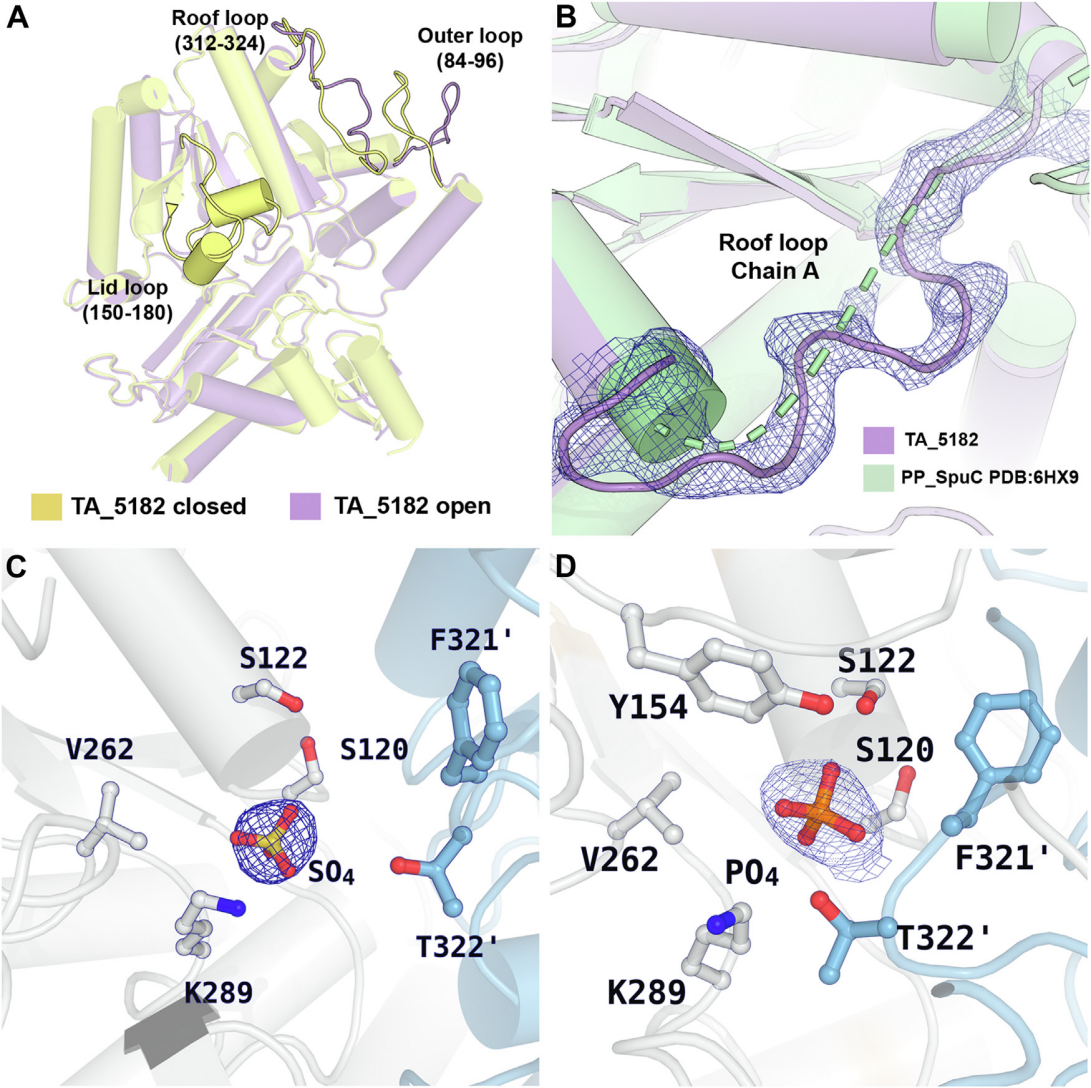
**Insights into the open and closed structures of TA_5182.***A*, superimposition of the monomers of the open (*purple*) and closed (*yellow*) crystal structures of dimeric TA_5182 shows the differences in the conformation of the loops in the enzyme. The electron densities of the loops in both the conformational states of the enzyme are shown in Fig S8. The overall conformational changes in the dimer can be visualized in Media S4. *B*, superimposition of the open state of TA_5182 (*purple*) and the putrescine TA from *P. putida* KT2440 (PDB ID: 6HX9) (*green*), showing the *2F*_*o*_*-F*_*c*_ electron density map contoured at 1.0 σ level (*blue mesh*) of the roof loop in TA_5182. *C*, active site architecture of TA_5182 in the open state with sulfate ion electron density found at the phosphate group binding cup. *D*, the active site architecture of the closed state of TA_5182 with phosphate ion bound the phosphate group binding cup of the enzyme. The 2*F*_*o*_*-F*_*c*_ electron density map of the phosphate and sulfate ions are represented in *blue mesh* (contour level: 1σ). The prime mark indicates the residues from another subunit of a dimer.

There are two highly flexible loops present in the structures of TA_2799 and TA_5182 (Fig. 2, *B* and *C*) that mediate the entry and exit of the cofactor and the substrate/byproduct into the active site of the enzyme, leading to significant conformational changes in the enzyme structure. Other than these two loops, there is another “outer loop” consisting of residues 84 to 96 that also shows conformational flexibility. We were able to crystallize TA_5182 in two different conformations which we term as the open and closed state (Fig. 3*A*). On formation of the internal aldimine with the PLP molecule, the enzyme shifts from its open state to a more compact closed form. To date, there are very few studies where similar conformational differences have been reported, such as in the transaminase from *C. violaceum* (11) and *Vibrio fluvialis* (16). In most of the apo form structures of the enzyme reported, the “roof” region (residues 312–321 in TA_2799) that lines up the substrate entry channel is not visible due to high flexibility of these regions. Comparing the structures of ω-TAs deposited in PDB shows that the roof structure is missing in the apo structures of fold type I transaminase enzymes from *Pseudomonas strains* (PDB ID: 5TI8, 6HX9) (17, 23). In our crystal structure of the apo state of TA_5182, the electron density of the roof loop is visible (Fig. 3*B*). In the crystal structure of the open state of TA_5182, the “lid” loop could not be visualized due to the inherent flexibility of this region. The “roof” loop projects itself toward the PLP-binding pocket on the binding of PLP in the active site of the enzyme. A sulfate ion can be observed at the PLP group phosphate binding cup (PGBC) of the active site of this enzyme (Fig. 3*C*). In the crystal structure of the closed state of TA_5182, electron density for the initial 32 residues and as well as that of the “roof” and “lid” loops are clearly visible. The conformation of the active site is similar to PLP-bound structures of ω-TAs; however, a phosphate ion can be seen at the PGBC of the enzyme (Fig. 3*D*).

TA_2799 was crystallized in the PLP-bound state. The conformation of the enzyme is almost identical to the closed state of TA_5182. Continuous high-quality electron density could be seen for the covalently bound internal aldimine form of the PLP bound to the catalytic K286 residue (Fig. 2*D*) in both the subunits. The TA_2799_L322F mutant was crystallized in its apo form, with a sulfate ion bound in the phosphate-binding pocket of the enzyme (Fig. 2*E*). These crystal structures and their analysis provide valuable information about the role of the different residues in and around the active site that mediate cofactor binding and the transamination reaction. More details about the roles of the active site residues and structural features of these ω-TAs in the catalytic reaction are discussed in the subsequent sections.

Superimposition of the closed state of TA_5182 and TA_2799 revealed that the former is almost identical to the PLP-bound state of the enzyme, with an RMSD of 1.107 Å over 741 Cα-atoms. On comparing the individual chains of the enzymes with other fold type I ω-TAs belonging to class III PLP-dependent enzymes, we found that the overall fold of TA_5182 and TA_2799 is conserved; however, the closest homolog of TA_2799, an ω-TA from *Pseudomonas jensenni* shares only 58% sequence identity. Comparing the open state of TA_5182 with the deposited structures in PDB resulted in an average RMSD of 2.0 Å, which is expected as the conformation of this state is significantly different than the PLP-bound state of the enzyme. The superimpositions of the monomers of TA_2799, TA_5182 open state, and TA_5182 closed state show that the monomers have an average RMSD of 0.153 Å, 0.166 Å, and 0.083 Å, respectively (Fig. S1 and Table S1). The major conformational differences can be found in the roof loop and the outer loop in the open state of TA_5182. A comprehensive list of the structural superimposition of the crystal structures of TA_2799 and TA_5182 with deposited structures of ω-TAs from other organisms found in the PDB, as calculated from the Dali server (http://ekhidna2.biocenter.helsinki.fi/dali/) is presented in Tables S2 and S3. The PLP-bound state of TA_2799 is similar to most of the structures found in the PDB, which is the catalytically competent state of the enzyme. The open state of TA_5182 is not easily observed. It shows a distinct conformation in the overall ternary structure of the enzyme, which allows the cofactor molecule and the substrates to enter the enzyme’s active site *via* movement of the flexible loops.

### Comparison of the active sites of TA_2799 and TA_5182

The high-resolution crystal structure of PLP bound TA_2799 provides insights into the architecture of the active site of this ω-TA. The active site is located in a deep cleft formed by the two monomers. Each dimer has two identical active sites which are formed by the contributions of the residues from both the subunits. The PLP molecule is linked to the ε-NH_2_ group of the catalytic K286 *via* a covalent Schiff base, and the pyrimidine ring of the cofactor molecule is sandwiched between the Y150 and I259 residues. The aromatic ring of the Y150 exhibits an edge-on interaction with the pyrimidine ring of the PLP molecule. The protonated pyrimidine nitrogen is stabilized by hydrogen bonded interactions with D257, and the hydroxyl group of the ring forms a water mediated hydrogen bond with E224. The phosphate group of PLP is stabilized by an intricate hydrogen bonding network, either directly or *via* water molecules, to S118, S285, Y150, and T323ʹ similar to the “phosphate group binding cup (PGBC)” described by Denesyuk *et al.* (24).

**Figure 4:**
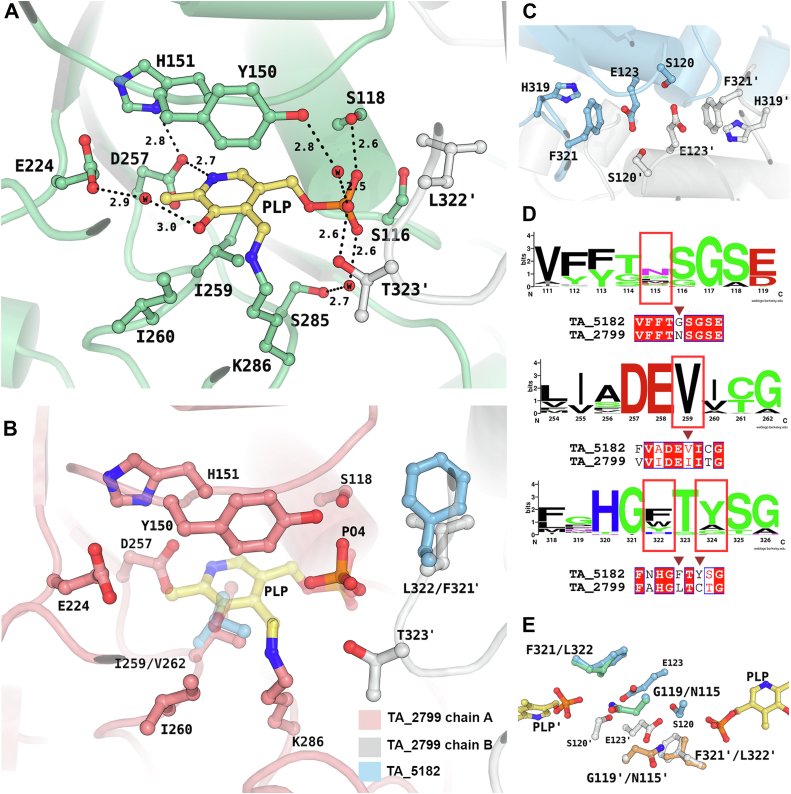
**Comparison of the active sites of TA_2799 and TA_5182.***A*, active site residues in PLP-bound state of TA_2799 showing water mediated polar interactions between the residue sidechains and PLP. The PLP molecule is covalently bound to the catalytic K286. The *black dotted lines* represent the polar interaction distances in Å. *B*, superimposition of TA_2799 and the closed state of TA_5182 showing the differing residues (*light blue*). The chains from the different monomers of TA_2799 are colored in *salmon* and *white*. The PLP molecule present in TA_2799 is colored in *yellow*, while the phosphate ion from TA_5182 is colored in *orange*. The phosphate ion binds at the phosphate binding cup of the enzyme. *C*, residues present at the dimeric interface of TA_5182 showing the E123 mediated anion-pi stacking interaction with F321. *D*, sequence logo representation of the conserved amino acids at the active site of bacterial ω-transaminases found in the PDB. The residues of interest are highlighted with red boxes. Residue numbers correspond to TA_2799 sequence. The sequence alignment of TA_2799 with TA_5182 for the highlighted residues are also depicted. *E*, Superimposition of TA_2799 and the closed state of TA_5182 showing the position of N115 in TA_2799 and F321ʹ in TA_5182. The N115 mediates an inter-subunit hydrogen bonding network in TA_2799. In TA_5182, a glycine is present instead of the asparagine. The prime mark indicates the residues from another subunit of a dimer.

The enzymes TA_2799 and TA_5182 have a sequence identity of 38.49% (Fig. S2), but the key residues that interact with the PLP molecule are identical except for I259 and L322 (Fig. 4, *A* and *B*). Comparison of TA_2799 with other known omega transaminases (10, 11, 14, 16, 17, 22, 25, 26) in the literature shows the presence of a conserved valine at the I259 position of TA_2799 (Fig. 4*D* and Data S1). The presence of a slightly bulkier I259 may play a significant role in the occupancy of the external aldimine molecule in the active site pocket and might be the reason for the rapid conversion of PLP to PMP by the enzyme. On the other hand, the L322 position in TA_2799 is usually occupied by an aromatic amino acid (Fig. 4*D*). In TA_5182, the position is occupied by F321, and the aromatic ring of phenylalanine contributes to an NH-π stacking interaction with an adjacent histidine residue, and an anion-π stacking with a conserved glutamate E123 on the other side, which confers stability to the dimer interface (Fig. 4*C*). Furthermore, in TA_2799, the glutamate residue interacts with an asparagine and a conserved serine residue to form an intricate hydrogen bonding network at the dimer interface. In TA_5182, the asparagine is replaced by a glycine residue (Fig. 4*E* and Data S1).

To assess the role of these residues in the cofactor binding and stability of omega transaminases, we constructed single mutants of TA_2799 and TA_5182. We compared the activity of the single mutants TA_2799_I259V, TA_2799_L322F, and TA_5182_G119N with the wild-type enzymes to increase the stability and the affinity of the enzymes toward its cofactor PLP. Apart from this, we also evaluated the role of the Y323 in TA_5182, which is also somewhat conserved in ω-TAs (Fig. 4*D*) by evaluating two mutants, TA_2799_L322F_C324Y and TA_5182_Y323C.

### Biochemical characterizations of the TA_2799, TA_5182, and their mutants

ω-TA enzymes have been widely used for biocatalysis of several unnatural substrates; however, the biotransformation of (*R*)-PAC to (*1R*, *2S*)-NE by ω-TAs is still poorly studied. The study by Sehl *et al.* (9) reports that other than the two enzymes discussed in this study which show good conversion of (*R*)-PAC, the TA from *C. violaceum* (CV2025) showed 96% activity against (*R*)-PAC, while the ω-TA from *P. aeruginosa* (PAO221) showed 38% activity against *(R)*-PAC. This study suggested that TA_2799 and TA_5182 can be utilized for efficient biocatalysis of (*R*)-PAC to (1*R*, 2*S*)-NE. To biochemically assess the activity of the TA enzymes TA_2799 and TA_5182 toward conversion of (*R*)-PAC to (1*R*, 2*S*)-NE, we used the triphenyl tetrazolium (TTC)-based assay developed by Sehl *et al.* (9). For kinetic studies, pyruvate was used as the amine acceptor and (*S*)-MBA was used as the amine donor. TA_2799_I259V, surprisingly, had very little activity (∼20%) compared to wild-type TA_2799 (Fig. S3).

**Figure 5:**
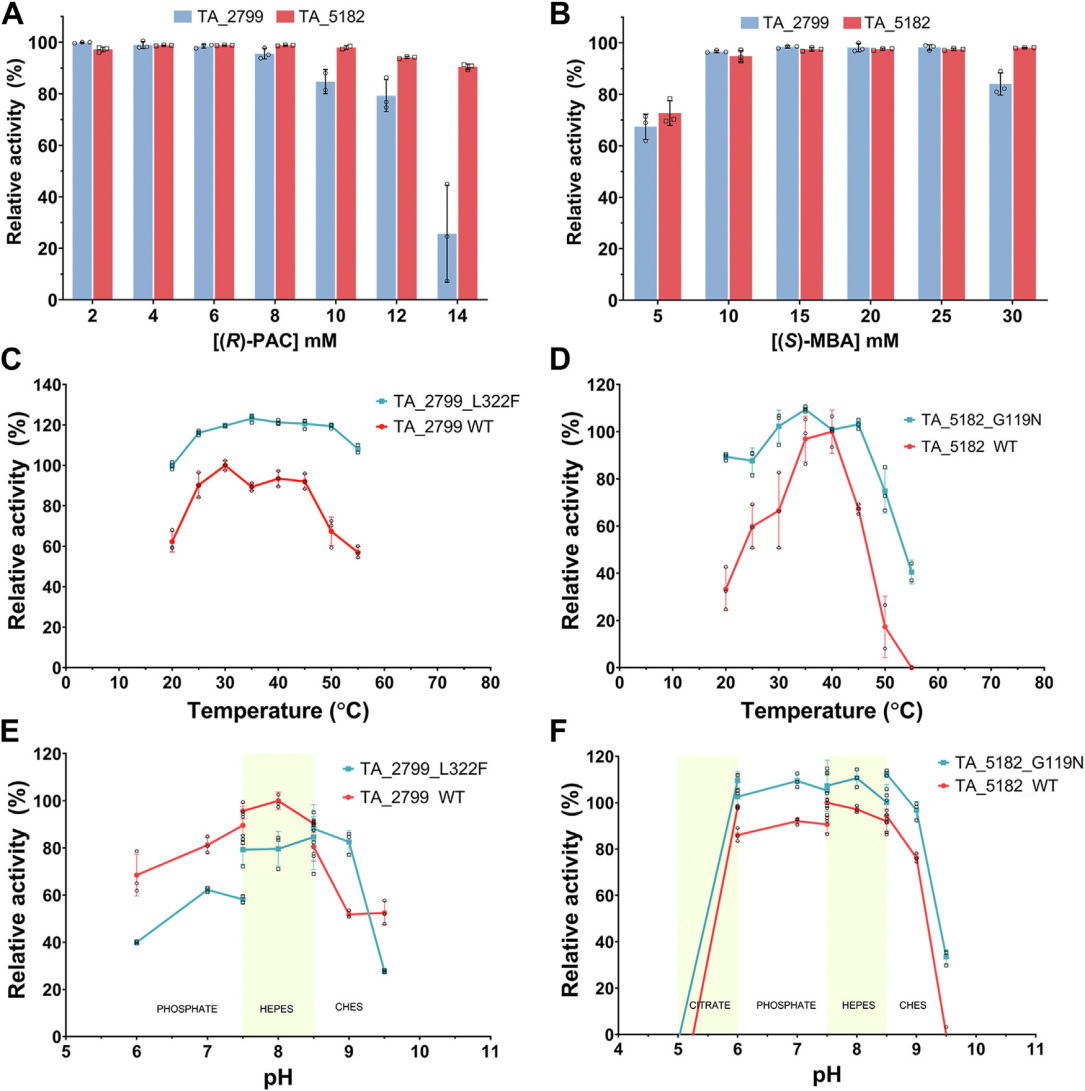
**Biochemical properties of TA_2799, TA_5182, and their mutants.***A*, comparison of the conversion of varying (*R*)-PAC concentration by TA_5182 and TA_2799 in the presence of PLP with (*S*)-MBA as the amino donor. *B*, conversion of (*R*)-PAC by TA_5182 and TA_2799 in the presence of PLP with varying concentrations of (*S*)*-*MBA. In both cases, efficient conversion of (*R*)-PAC occurs with a minimum donor: acceptor ratio of 2:1. *C*, temperature optimum curve of TA_2799 and TA_2799_L322F. The mutant enzyme shows >100% activity over a broad temperature range compared to its wild-type counterpart and is active at 50 °C as well. *D*, temperature optimum curve of TA_5182 and TA_5182_G119N. The mutant maintains 100% activity at 50 °C and maintains ∼80% activity over a wide range of temperature. *E*, pH optimum curve of TA_2799 and TA_2799_L322F. The pH optimum in the mutant enzyme is shifted to pH 8.5, while the wild-type enzyme shows optimum activity at pH 8.0. *F*, pH optimum curve of TA_5182 and TA_5182_G119N. The mutant enzyme follows a similar trend, but shows increased activity compared to the wild-type enzyme. Each assay was performed in triplicates, individual datapoints are denoted by a ◦ symbol. Error bars represent standard deviation of mean.

The TA enzymes are known to demonstrate substrate inhibition (27). To understand the extent of substrate concentration that can be used for the TA enzymes for efficient catalysis, the enzymes were subjected to varying concentrations of the substrates, keeping other parameters fixed. It was observed that TA_5182 has a better tolerance towards substrate inhibition in comparison to TA_2799. Interestingly, the ratio of the amino donor: amino acceptor needs to be at least 2:1 for efficient catalysis (>90% conversion of (*R*)-PAC) while using (*S*)-MBA as the amino donor (Fig. 5, *A* and *B*).

We studied the effect of temperature on the activities of the wild type and mutant enzymes. The TA_2799_L322F mutant showed a 20% increase in enzymatic activity for the mutant variant across a range of temperatures compared to the wild-type enzyme (Fig. 5*C*). The optimum activity for the wild-type TA_2799 enzyme was observed at 30 °C, while the TA_2799_L322F mutant maintained 100% activity across a wide range of temperatures from 25 to 55 °C. For TA_5182 and the TA_5182_G119N mutant, a similar temperature profile was observed (Fig. 5*D*). The G119N mutant showed optimum activity at 30 °C and maintained more than 60% activity at 50 °C as compared to the wild-type enzyme, whose activity dropped to 20%.

The pH-dependent activities of the enzymes and their mutants were also measured. It was found that TA_2799 exhibits the highest activity at a pH range of 7.5 to 8.5. The pH optima tend to shift toward alkaline pH (pH 9.0) in the case of the L322F mutant (Fig. 5*E*). TA_5182, on the other hand, was found to be active in a broader pH range. The optimum activity of the enzyme was found to be at pH 7.5, but it retained ∼80% of its activity at pH 6.0 as well. The activity dropped drastically to zero at pH 5.0 (Fig. 5*F*). A similar trend was observed for the G119N mutant as well. Notably, the mutant enzyme showed a 10% increase in relative activity compared to its wild-type counterpart.

### Cofactor release and PLP-dependent conversion of (*R*)-PAC by **ω**-TAs

Loss of the aminated cofactor (PMP) formed during the first half of the reaction is a major cause for inactivation of transaminases (10, 27). A study by Schell *et al.* (28) showed that the PMP generated by the TA enzymes is released into the reaction medium due to the flexibility of the loops, as PMP is no longer covalently bound to the enzyme.

**Figure 6:**
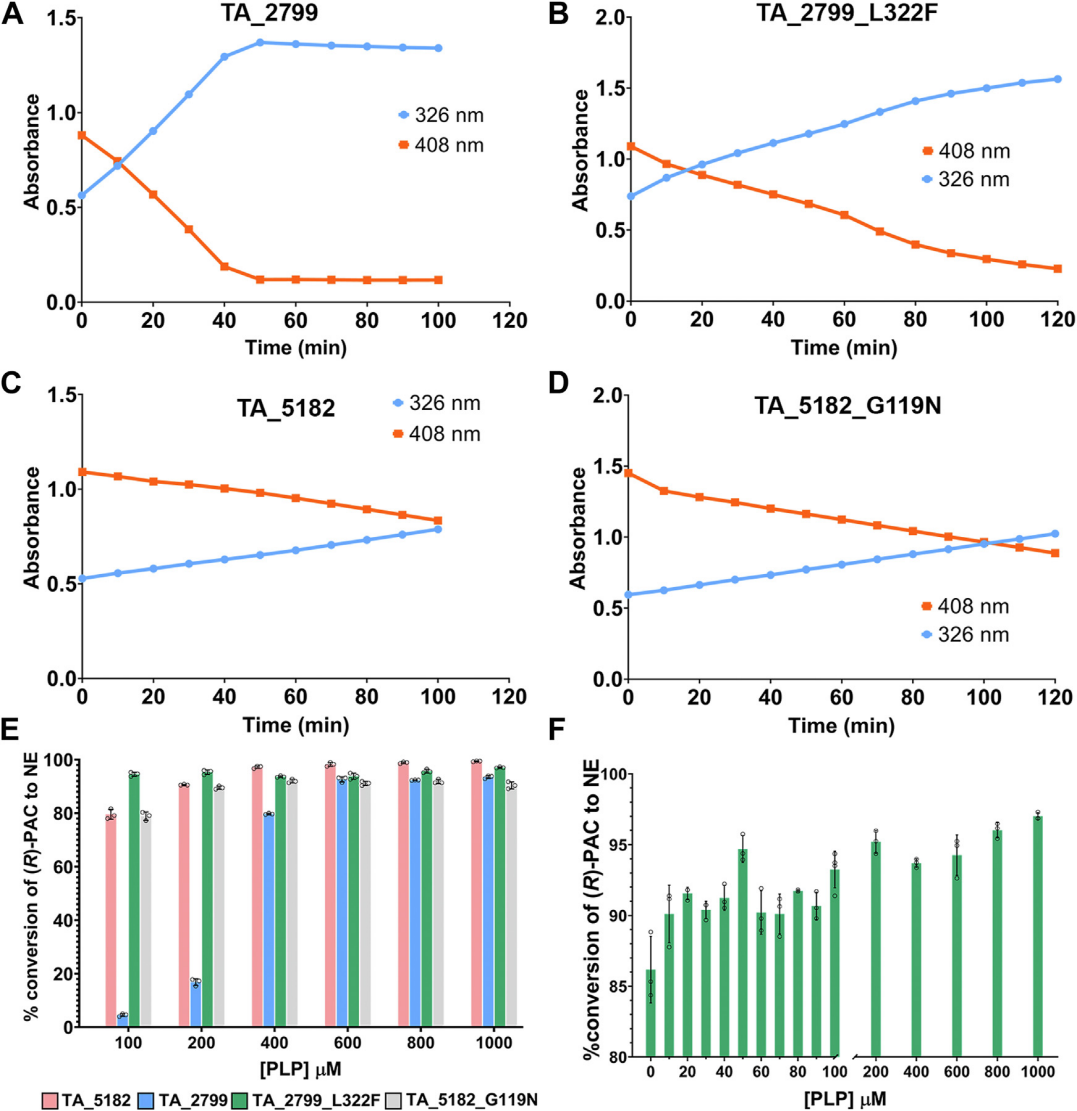
**Time-dependent conversion and PLP-dependent enzyme activity of TA_2799, TA_5182, and their mutants.***A*, time-dependent conversion of PLP to PMP by enzymes incubated with 1 mM PLP and 10 mM *(S)*-MBA (amine donor) by TA_2799, (*B*) TA_2799_L322F, (*C*) TA_5182, and (*D*) TA_5182_G119N. *Orange line* represents the absorbance at 408 nm that denotes the amount of free PLP in solution, while the *blue line* denotes the absorbance of free PMP at 326 nm. Compared to the wild-type, TA_2799, the L322F mutant shows a slower rate of PMP release. Each reaction was performed in triplicates, however only one dataset is plotted for clarity. *E*, conversion of *(R)-*PAC in the presence of *(S)-*MBA by the TA enzymes at varying concentrations of PLP. For efficient conversion of (*R*)-PAC, TA_2799 requires at least 600 μM of PLP to be supplemented externally to the reaction. *F*, conversion of *(R)-*PAC in the presence of *(S)*-MBA by TA_2799_L322F at varying concentrations of PLP. The mutant enzyme exhibits ∼85% activity even without any external addition of PLP in the reaction. Each assay was performed in triplicates, individual datapoints are denoted by a ◦ symbol. Error bars represent standard deviation of mean.

To investigate the cofactor release TA_2799 and TA_5182 were incubated with 1 mM of PLP and 10 mM of (*S*)-MBA and incubated at 30 °C in the dark in the presence and absence of 5 mM amine acceptor (*R*)-PAC. PLP in solution has an Absorption maximum (Abs_Max_) at 408 nm and PMP has an Abs_Max_ at 326 nm. The spectra showed characteristic peaks at 408 nm and 326 nm for PLP and PMP, respectively. The shorter roof region of TA_5182 was thought to be more flexible and thus caters to escape from the cofactor. However, when (*R*)-PAC was not present in the solution, it was seen that TA_2799 converted all the PLP into PMP, while TA_5182 still retained some of the PLP in the solution (Fig. 6, *A* and *C*). TA_2799 carries out this conversion much faster compared to TA_5182. There was no change in the PLP release profile of the TA_5182_G119N mutant (Fig. 6*D*). In the cofactor binding pocket of TA_2799, as mentioned earlier, there are substitutions at the I259 in place of valine found in other ω-TAs. The presence of the bulkier isoleucine residue may play a significant role in the release of the unbound external aldimine PMP from the cofactor binding pocket, thus leading to rapid conversion of PLP in the reaction mixture. However, the mutant TA_2799_L322F shows conversion of PLP to PMP at a slower rate than the wild-type enzyme, which indicates that F322 has a key role in retaining the PMP to the enzyme active site when the covalent bond has been broken (Fig. 6*B*).

The conversion of 5 mM (*R*)-PAC in the presence of 10 mM (*S*)-MBA at different concentrations of the cofactor PLP was also studied. TA_5182 showed efficient conversion at PLP concentrations of >200 μM, while TA_2799 required >400 μM of the cofactor in the reaction mixture for >80% conversion of (*R*)-PAC (Fig. 6*E*). The TA_2799_L322F mutant was probed for its PLP dependency. Interestingly, this mutant shows exceptionally good conversion efficiency, with >90% conversion of (*R*)-PAC even at PLP concentrations lower than 100 μM (Fig. 6*F*).

### Storage stability of the transaminases

**Figure 7:**
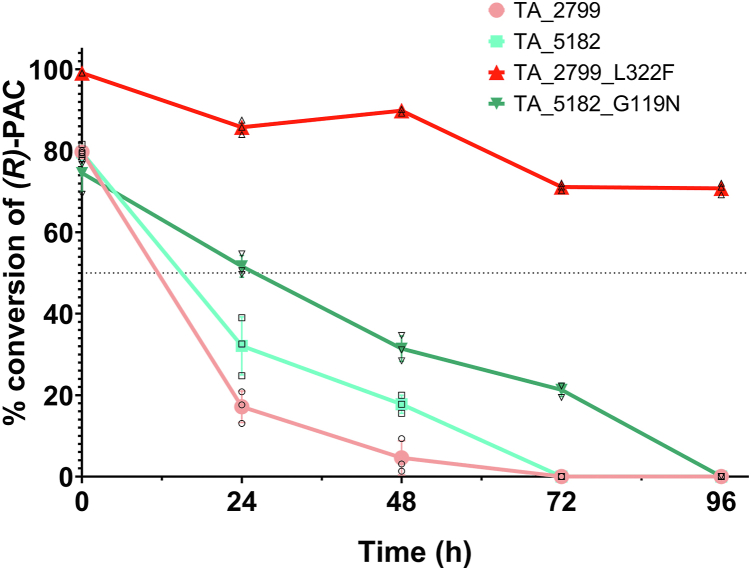
**Conversion of *(R)*-PAC by TA_2799, TA_5182 and the mutant enzymes after storage at 30 °C for different time periods**. The mutant enzymes retain their activity better than their wild-type counterparts. The TA_2799_L322F mutant retains ∼70% of its activity even after 96 h of storage at 30 °C. Reactions were performed in triplicates. Individual datapoints are denoted by their corresponding symbol. Error bars represent standard deviation of mean.

The transaminase enzymes were stored at 30 °C without any external addition of PLP to measure the storage stability of the enzymes. TA_2799 lost its activity within 24 h, while TA_5182 retained ∼40% of its activity for 48 h. The TA_5182_G119N mutant showed better tolerance compared to its wild-type counterpart, as it retained 20% of its activity after 72 h of storage. The TA_2799_L322F mutant showed the best increase in stability; it retained ∼70% of its activity even after 96h of storage (Fig. 7). A possible explanation is that the mutant enzymes can capture the PLP cofactor from the cell lysate during the process of purification (as observed from the yellow coloration), which helps them maintain their tertiary structures. The binding of the PLP molecule at the PLP binding site reduces the conformational dynamics in the enzyme, as a result, the irreversible unfolding process is hindered.

### Kinetic parameters of the enzymes and their mutants

We evaluated the kinetic parameters of the TA enzymes and their mutants, as reported in Table 2. ω-TA enzymes are slow in their catalysis of unnatural substrates (26). It was seen that TA_2799 was ∼15 times faster than TA_5182 in terms of their rates of catalysis, although the affinity of TA_5182 for the amine donor substrate *(S)*-MBA was ∼6-fold greater than that of TA_2799. The mutant TA_2799_L322F showed an increased affinity toward the amine donor; however, the *V*_max_ of the enzyme and the turnover number were reduced, probably because of the diminished movement of the dimeric interface. However, the catalytic efficiency of the mutant enzyme was calculated to be slightly greater than its wild-type counterpart. The TA_5182_G119N mutant also showed ∼3-fold increased affinity for the amine donor (*S*)*-*MBA compared to the wild-type TA_5182; however, the turnover number of the mutant enzyme was reduced. However, the catalytic efficiency of the mutant enzyme was slightly enhanced due to the increased affinity toward the substrate (Table 2).

**Table 2:** Kinetic parameters of the ω-TA enzymes against the amino donor (*S*)-MBA

Enzyme	*K*_m_ (mM)	*V*_max_ (U mg^−1^)	k_cat_ (min^−1^)	k_cat_/*K*_m_ (mM^−1^ min^−1^)
TA_2799	6.459 ± 0.621	0.104 ± 0.007	4.321 ± 0.279	0.668
TA_2799_L322F	1.068 ± 0.566	0.042 ± 0.001	1.777 ± 0.036	1.664
TA_5182	1.089 ± 0.117	0.007 ± 0.0002	0.294 ± 0.007	0.269
TA_5182_G119N	0.347 ± 0.024	0.003 ± 0.0005	0.1468 ± 0.002	0.423

## Discussion

Large-scale industrial applications of wild-type transaminases are mostly limited by operational instability and enzyme inactivation (10). Different approaches towards engineering wild-type transaminases have shown promise for increasing the yield of the desired amine product and improving the stability of the enzyme (29). The dependence of the enzyme on the PLP cofactor limits the applicability of these enzymes since external addition of the cofactor increases production costs (14). Moreover, cofactor binding enhances the stability and the catalytic activity of the enzymes (15). Our study hints at the possible factors that can lead to the development of ω-TAs with enhanced affinity for the cofactor as well as improved thermal stability of the engineered enzymes. Taking into account the structural and biochemical data on TA_2799, TA_5182 and their variants, we discuss the importance of some of the structural features that modulate the functional properties of ω-TAs.

### Loop movement and structural orientation of the F321 in TA_5182

**Figure 8:**
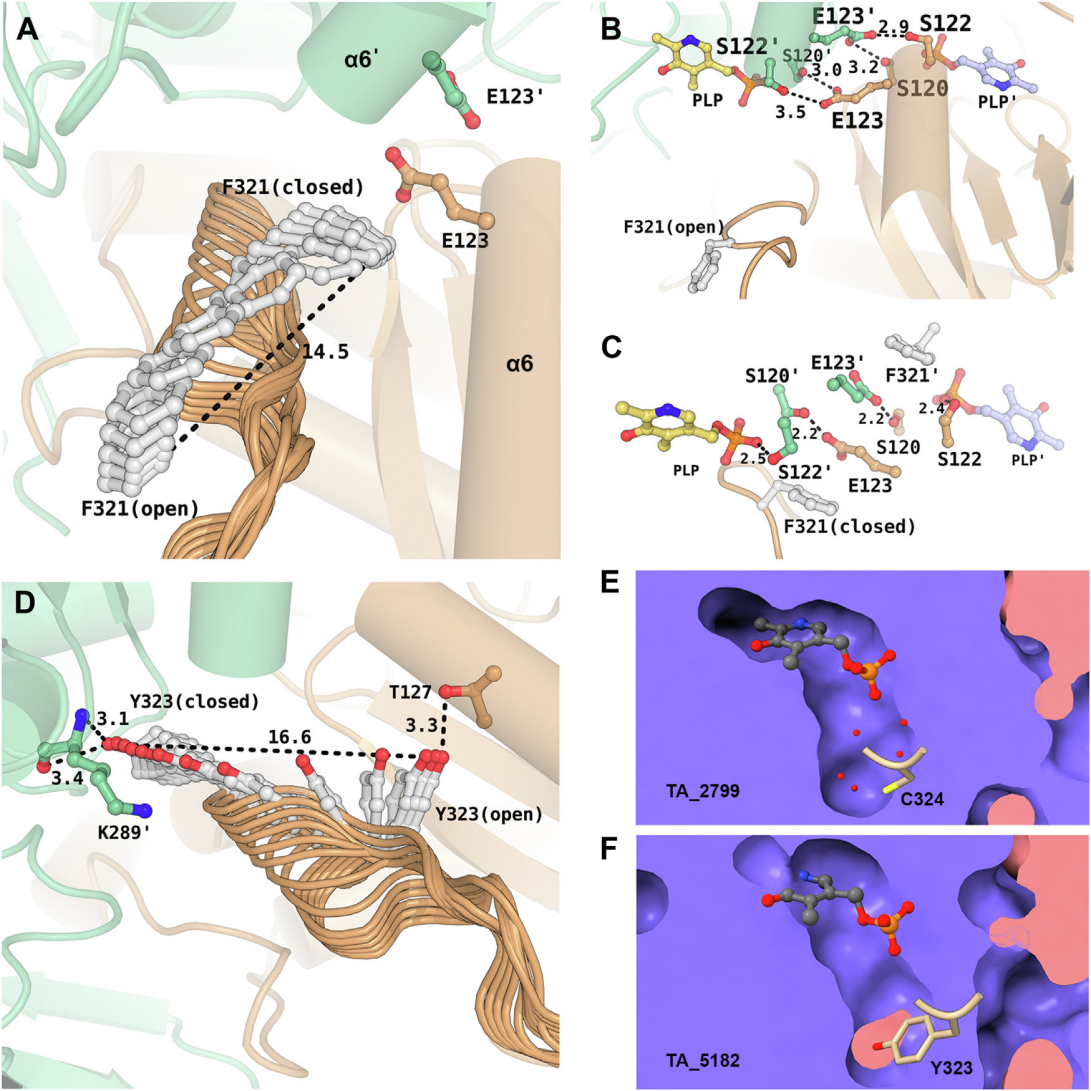
**Loop movements in TA_5182.***A*, movement of F321 in TA_5182 from open to closed state. The E123 residue from each monomer is shown in ball and stick representation. The *black dotted line* represents the displacement of the F321 residue from open to closed state. *B*, interactions between E123, S120 and S122 of the two subunits in the open state of TA_5182 show the orientation of the hydroxyl (-OH) group of S122 away from the PLP binding pocket. *C*, interactions between E123 and S120 of the two subunits in the closed state of TA_5182. S122 is no longer in hydrogen bonding distance with E123 and is free to interact with the phosphate group of the PLP molecule. The PLP molecule has been superposed from the crystal structure of TA_2799. *D*, movement of Y323 in TA_5182. Interactions between Y323 and T127 at initial open position. In the closed state, Y323 interacts with the main chain of K289ʹ, probably playing a role in stabilization of the residue during catalysis. All distances are presented in Å. *E*, PLP crater of TA_2799. *F*, PLP crater of TA_5182 The tyrosine residue fills the crater. The prime mark indicates the residues from another subunit of a dimer. All the figures are made in PyMol. The trajectory of the loop movement has been generated using the “morph” command in PyMol over 60 steps with a refinement parameter of 10.

The F321 (L322 in TA_2799) in TA_5182 is present in the highly flexible loop, also known as the “roof” of the PLP binding site (11). This loop projects into the PLP binding pocket upon PLP binding in the enzyme active site. Most apo-crystal structures of ω-TAs have been determined with either phosphate or a negatively charged ligand at the phosphate binding pocket of the active site of the enzyme. As described by Humble *et al.* in their study of ω-TA from *C. violaceum* (11), the position of the loop is vastly different in the one of the apo states of the enzyme (PDB ID: 4A6U), where the roof and lid loops show high flexibility. In the apo-open state, the F321 in TA_5182 is initially positioned near the surface of the enzyme, widening the substrate entry channel of the enzyme. On PLP binding, this residue moves by ∼14 Å, orienting toward the α6 helix, which has S120 and S122 from the other subunit (Fig. 8, *A* and *B*). This restructuring shifts S120 and S122 from their initial positions, breaking their interactions with E123ʹ, allowing S120 to coordinate the phosphate group of PLP (Fig. 8*C* and Media S2). The F321 becomes stabilized through π-stacking interactions, sandwiched between the neighboring H319 and the E123 (Fig. 4*C*). In TA_2799, the side chain of L322 is not bulky enough to cause this change, and as a result, the phosphate group of PLP may not be as tightly coordinated when the PLP molecule is bound to the enzyme. L322 cannot form stacking interactions with H319 and G123 like aromatic amino acids. The effect of the L322F mutation is observed in the thermal stability assay, as the mutant enzyme has an increased ∼6 °C melting temperature (Tm_app_) over the wild-type TA_2799. The mutant enzyme’s affinity towards the cofactor PLP is clearly enhanced as observed by the yellow coloration of the enzyme solution post-purification and the characteristic peak for PLP at 420 nm seen in the absorbance spectra of the purified enzyme, similar to the report of Kwon *et al.* (30) (Figs. S4*A* and S5*A*). The mutant enzyme efficiently converts (*R*)-PAC to (1*R*, 2*S*)-NE even without external cofactor addition to the reaction mixture. We expected PLP electron density in the crystal structure of the TA_2799_L322F mutant; however, only sulfate ion density could be seen. PLP may have been present at the active site, but not covalently bound as an internal aldimine, and thus, the sulfate ions from the crystallization mother liquor could compete and displace the PLP molecule out of the active site pocket.

### Evaluating the role of conserved Y322 in TA_5182 in stabilizing PLP

In the same F321 roof loop, the adjacent Y322 of TA_5182 was also observed for its orientation after PLP binding. Y322 is initially in a hydrogen bond with T127. As the loop rearranges, Y322 moves ∼16 Å to position itself just below the PLP molecule, and the hydroxyl group of Y322 interacts with the main chain of the catalytic K289ʹ, stabilizing the catalytic residue (Fig. 8*D* and Media S3). This orientation of Y322 creates a floor beneath the PLP molecule, covering the deep crater and allowing better orientation of the PLP molecule to form the internal aldimine. In TA_2799, C324 occupies the same position. C324 is not adequate to cover the crater, so the crater in the crystal structure of TA_2799 is filled with solvent molecules instead (Fig. 8, *E* and *F*).

Based on this observation, we generated the TA_2799_L322F_C324Y double mutant. However, this mutant showed no yellow color after purification, indicating that the affinity of this double mutant enzyme towards PLP was significantly reduced in comparison to the single mutant 2799_L322F. The enzyme precipitated within 72 h of incubation at 4 °C without added cofactor or glycerol. The *K*_M_ of the double mutant for (*S*)-MBA was 1.188 ± 0.122 mM, which indicates slightly decreased affinity of this mutant for the amino donor substrate compared to the L322F mutant. The *V*_max_ and *k*_cat_ values also decreased to 0.033 ± 0.001 U mg^−1^ and 1.381 ± 0.049 min^−1,^ respectively. There was a slight increase in the *k*_cat_/*K*_M_ (1.162 mM^−1^ min^−1^) in comparison to the wild-type TA_2799 (Table S4).

Since this mutation did not increase the affinity of the enzyme for PLP, we mutated the enzyme TA_5182 at position 323, which is typically a conserved tyrosine or phenylalanine in known bacterial transaminases, to cysteine as found in TA_2799. However, this mutant showed no detectable activity against (*R*)-PAC and thus was not studied further for its implication in PLP binding.

### Role of N119 in PLP binding in TA_5182

**Figure 9:**
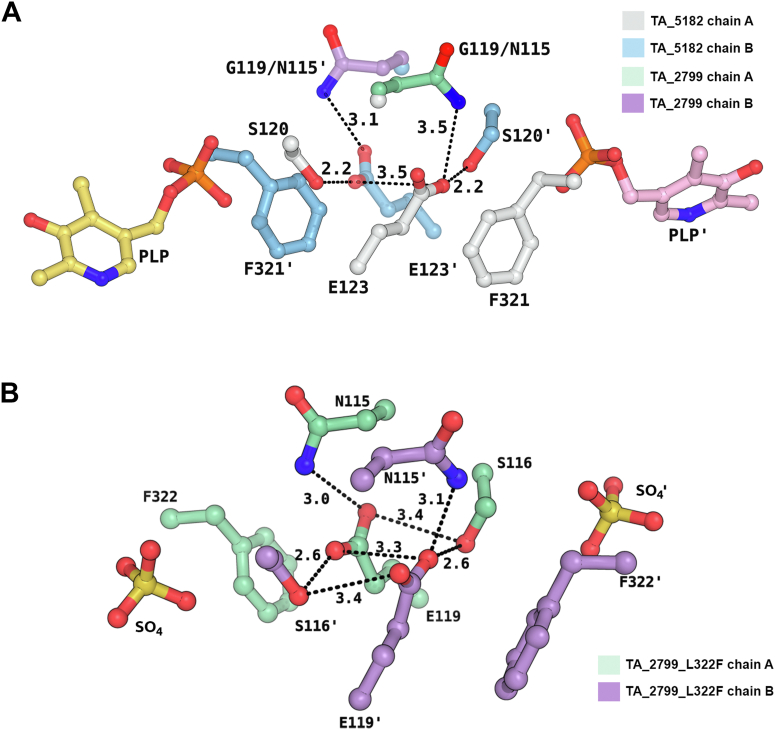
**Intersubunit hydrogen bonding network influences dimer stability of ω-TAs.***A*, superimposition of TA_2799 and TA_5182 in its closed state showing possible hydrogen bonding network between the sidechains of S120, E123 and N115. The chains A and B of TA_5182 are colored in *light-blue* and *light-gray*, respectively. The N115 of TA_2799 from chains A and B are colored in green and purple respectively. In contrast, TA_5182 contains a glycine at the 119th position. The PLP molecule represents the position of the active site of the enzymes. *B*, hydrogen bonding network between the sidechains of S116, E119 and N115 in TA_2799_L322F mutant. The chains A and B are colored in *green* and *purple* respectively. The sulfate ions represent the position of the PGBC of the enzyme. The prime mark indicates the residues from another subunit of a dimer.

Along with the conserved F321 and E123 residues that form an inter subunit network, the presence of the asparagine residue plays a key role in increasing the affinity of the enzyme towards its cofactor (Fig. 9, *A* and *B*). The F321 (L322 in TA_2799) is conserved in many annotated ω-TAs, but current reports do not describe yellow color formation due to the presence of PLP in the active site of the purified enzyme. TA_5182 has phenylalanine at the same position but shows a slight yellow color at concentrations >30 mg/ml. We were interested to understand the reason behind the enhanced affinity of the 2799_L322F mutant towards the cofactor. In the crystal structure of the TA_2799_L322F mutant enzyme, the ND1 of the N121 residue maintains hydrogen bonds with both OE1 and OE2 of E123, which provides further stability to the anion-pi stacking interaction between E123 and F322 in the same monomer. Apart from that, the OE1 of E123 forms a hydrogen bond with OG of S120, forming an extensive intersubunit network that stabilizes the dimer (Fig. 9*B*). In the crystal structure of the ω-TA from *C. violaceum* (PDB ID: 4A6T) and *V. fluvialis* (PDB ID: 4E3R), a similar network is seen. The authors reported that the purified protein appears yellow in solution, supporting our observation. Evaluation of reported structures of ω-TAs from various *Pseudomonas* sp. revealed that the ω-TA from *P. jensenni* (PDB ID: 6G4B) harbors an asparagine at this position, and has a Tm_app_ of 62 °C, quite similar to the TA_2799_L322F mutant (26). All other reported ω-TAs from other *Pseudomonas* strains, like *Pseudomonas* str. AAC (PDB IDs: 5TI8 and 4UHM) (23, 25), *P. aeruginosa* (PDB ID: 4B98) (22), and *P. putida* (PDB ID: 6HX9) (17) contain glycine at this position, like TA_5182. Introducing glutamine at position 119 in TA_5182 (replacing glycine) significantly increased the affinity of the enzyme TA_5182 toward PLP, as observed from the yellow coloration of the purified protein and spectral data (Figs. S4*B* and S5*B*). In the crystal structure of the ω-TA from *Pseudomonas fluorescens* (PDB ID: 6S54) (14), there is a leucine and aspartate instead of the asparagine and glutamine as seen in TA_2799. The authors reported that the V129N mutation at the PLP binding pocket does not increase the PLP affinity of the protein. A possible reason for such observation can be explained by our study, as the inter-subunit network as described in Figure 9*A* is missing in the TA enzyme from *P. fluorescens*.

## Conclusions

Our study on two ω-transaminases (ω-TAs) from *P. putida* KT2440 presents structural insights into the substrate and cofactor binding pocket to understand the molecular basis of PLP affinity of bacterial transaminases. We solved the high-resolution, cofactor-bound structure of TA_2799. We crystallized TA_5182 in two distinct conformations and resolved the structure of a highly flexible loop that plays a direct role in accommodation of the PLP cofactor in the active site of ω-TAs. This loop is not observed in most of the reported apo forms of the enzyme. The structural information obtained from these crystallographic models revealed key interactions between the residues around the cofactor binding pocket. The cumulative effects of these interactions enhance the binding of the PLP cofactor, which in turn stabilizes the overall fold and restricts the loop mobility. This is observed by the increase in the melting temperature of the mutants of both the enzymes, along with a significant enhancement in their enzyme activity with *(S)*-MBA and (*R*)-PAC as substrates. In TA_5182, F321 plays a crucial role in accommodating the phosphate moiety of the cofactor molecule. Additionally, we identified an asparagine residue (N115 in TA_2799) which participates in hydrogen bonding interaction and forms an intricate inter-subunit network that enhances the PLP affinity as well as the dimer stability of the transaminase enzymes. Collectively, the structural data, mutations and biochemical studies, the results of this study provide a detailed understanding of how to develop stable and efficient ω-TAs for biotransformation.

## Experimental procedures

### Lysis and elution buffers

Two types of buffers were prepared for protein purification. Lysis Buffer 1: 50 mM Sodium Phosphate (pH 8.0), 300 mM NaCl; Lysis Buffer 2: 50 mM HEPES (pH 8.0), 300 mM NaCl. The corresponding elution buffers for Ni-NTA affinity chromatography additionally contained 250 mM imidazole. TA_5182 was purified in Lysis Buffer 1 for setting up crystallization trays. For all other studies, Lysis Buffer 2 was used.

### Protein expression and purification

The genes encoding *P. aeruginosa* KT2440 ω-TAs TA_2799 and TA_5182 were inserted into pET43.1b expression vectors, downstream of the 6x-His Tag and transformed into *E. coli* BL21 (DE3) cells by heat shock. Overnight-grown *E. coli* cultures were transferred to 5 L baffled Erlenmeyer flasks containing 1.5 L of LB (Luria-Bertani) broth supplemented with 100 μg/ml ampicillin and induced with 300 μM isopropyl β-D-1-thiogalactopyranoside (IPTG) at OD_600_ = 0.9 for protein overexpression. Cells were harvested by centrifugation after 5 h of induction at 24 °C in mild shaking conditions (100 rpm), resuspended in lysis buffer (L1 or L2), and homogenized by sonication. The TA enzymes were purified *via* Ni-NTA affinity chromatography (HisTrap column, GE Healthcare) using the buffers described above. The eluted fractions were concentrated to 15 mg/ml and buffer-exchanged into the corresponding lysis buffers to remove imidazole.

### Sequence alignment and conserved residue analysis of TA_2799 and TA_5182

The sequences of TA_2799 and TA_5182 were procured from UniProt database and aligned using Clustal Omega (31). The conserved regions (highlighted in red) were analyzed using ESPript 3.0 web server (https://espript.ibcp.fr) (32). To analyze the conserved regions in bacterial fold type I ω-TAs, sequences of bacterial fold type I ω-TAs available in the PDB were assessed using Weblogo tool (https://weblogo.berkeley.edu) and Clustal Omega (33).

### Site-directed mutagenesis of TA enzymes

Each PCR reaction mixture consisted of DNA template (pET 43.1b-TA_2799/TA_5182, 60 ng), forward and reverse primers (10 μM, 6.75 μl each), 5X Q5 reaction buffer (10 μl), Q5 GC enhancer (10 μl), Q5 DNA Polymerase (NEB) (1 μl), and nuclease-free water to a final reaction volume of 50 μl. PCR was performed as follows: 98 °C for 10 min; 30 cycles of 98 °C for 1 min, 64 to 72 °C (annealing) for 30 s, and 72 °C for 10 min; final extension at 72 °C for 10 min 5 μl of 5X FastDigest Buffer (Thermo Fisher Scientific) was added to the PCR product and digested with 1 U of DpnI by incubation at 37 °C for 2 h. The digested product was used directly to transform competent *E. coli* DH5α cells *via* heat shock (2 min). The transformed bacteria were plated on LB agar supplemented with 100 μg/ml ampicillin and incubated overnight. Isolated colonies were grown in LB medium containing ampicillin, and plasmids were isolated using the Exprep Mini Plasmid Purification Kit (Genexy). Mutations were verified by sequencing, and *E. coli* BL21 (DE3) cells were transformed using purified plasmids. The primers used for the SDM studies are listed in Table S5.

The mutant enzymes were overexpressed and purified using the same procedure as described for the wild-type enzyme.

### Enzyme activity assay

The triphenyl tetrazolium chloride (TTC)-based assay reported by Sehl *et al.* (9) was used to investigate the activity of the TA enzymes and their mutants. An 80 μl reaction volume containing 6.25 mM (*R*)-PAC and 12.5 mM (*S*)-MBA was set up in reaction buffer (50 mM HEPES pH 8.0, 400 μM PLP). To this mixture, 20 μl of enzyme (10 mg/ml) was added, to achieve final concentrations of 5 mM amine acceptor ((*R*)-PAC) and 10 mM of amino donor ((*S*)-MBA) in a 100 μl reaction volume. For the standard curve, 100 μl aliquots of (*R*)-PAC in reaction buffer at concentrations ranging from 1 mM to 5 mM were prepared. All enzyme assays were performed in 96-well flat-bottom microtiter plates (Eppendorf). The plates were incubated in the dark for 2 h at 30 °C. After incubation, all the samples were subjected to 10-fold dilution with distilled water and 40 μl of TTC solution (1 mg/ml of TTC salt in a 1:3 v/v solution of 75% ethanol and 1 M NaOH) was added. The reaction of TTC with unreacted (*R*)-PAC led to the formation of a red tetrazolium salt, and its absorbance was measured at 510 nm using a spectrophotometer (VantaStar BMG LabTech). All assays were performed in triplicate.

To evaluate the substrate tolerance of the TA enzymes and their mutants, enzyme activity was measured at varying concentrations of either amino donor or the amino acceptor while keeping other parameters constant. To evaluate the efficiency of the enzymes toward (*R)*-PAC, enzyme activity was measured at varying concentrations of (*R*)-PAC at a fixed concentration of 25 mM (*S*)-MBA and 1 mM PLP. Likewise, the efficiency of the enzymes towards (*S*)-MBA was measured at varying concentrations of (*S*)-MBA at a fixed concentration of 5 mM (*R*)-PAC and 1 mM PLP.

Temperature-dependent conversion of (*R*)-PAC was measured at different temperatures ranging from 25 °C to 60 °C. The enzymes were incubated with the reaction mixture for 1.5 h, denatured by heating the assay mixture at 90 °C for 5 min, and flash-cooled on ice. The denatured enzyme was removed from the mixture by centrifuging at 10,000*g* for 3 min, and 10 μl of the supernatant was aliquoted for estimation of remaining (*R*)-PAC.

For pH-dependent studies, different buffers corresponding to a pH range from 5.0 to 9.5 were used to prepare the reaction mixtures containing 6.25 mM (*R*)-PAC, 12.5 mM (*S*)-MBA and 500 μM PLP. 20 μl of the purified enzyme (2 mg/ml) was added to each reaction and incubated for 1.5 h, denatured by heating the assay mixture at 90 °C for 5 min, and flash-cooled in ice. The denatured enzyme was removed from the mixture by centrifuging at 10,000*g* for 3 min, and 10 μl of the supernatant was aliquoted for estimation of remaining (*R*)-PAC.

For kinetic studies, the first half of the transamination reaction was measured using high-performance liquid chromatography (HPLC). (*S*)-MBA (0–5 mM) was used in the reaction mixture containing 20 mM pyruvate as amino acceptor, 2 mM PLP, and 200 μg of TA enzyme in 100 μl reaction buffer. After 30 min of incubation at 30 °C, the reaction was stopped by heating the mixture to 90 °C, and 400 μl of ddH_2_O was added to the reaction. The mixture was centrifuged at 16,000*g* for 20 min to remove the denatured enzyme. 10 μl of the supernatant was injected into the HPLC system (Jasco MD-4015) at a flow rate of 1 ml/min through an Agilent C-18 column. The mobile phase consisted of 30:70 (v/v) acetonitrile:ddH_2_O mixture, and the amount of acetophenone produced was measured at 245 nm. One unit (U) of enzyme activity denotes the amount of enzyme required to produce 1 μmol of acetophenone per minute.

### Time-dependent PLP conversion assay

To measure PLP conversion by the TA enzymes and their mutants, 100 μl reactions were prepared in HEPES buffer pH 8.0, 1 mM PLP and 10 mM (*S*)-MBA. 100 μg of enzyme was added per reaction. The assays were performed in 96-well flat-bottom microtiter plates (Eppendorf) and readings were taken at 10-min intervals, for 2 h. The spectra were recorded from 300 nm to 500 nm.

For PLP dependent conversion of (*R*)-PAC, 100 μl reactions were set up in 96-well flat-bottom microtiter plates (Eppendorf) consisting of 5 mM (*R*)-PAC, 10 mM (*S*)-MBA, 100 μg of enzyme and different concentrations of PLP ranging from 0 to 1000 μM. The plates were incubated at 30 °C for 4 h and the remaining (*R*)-PAC was measured using the TTC based method mentioned earlier.

### Storage stability assay

Freshly purified TA enzymes and their mutants were concentrated to 10 mg/ml and stored at 30 °C in the dark for 96 h. Conversion of 5 mM (*R*)*-*PAC in the presence of 10 mM (*S*)*-*MBA was measured at periodic intervals in 100 μl reactions as described earlier.

### Crystallization

The enzymes TA_2799, TA_5182 and TA_2799_L322F were concentrated to 15 to 20 mg/ml for setting up crystallization trials. In the case of TA_2799, additional glycerol at a final concentration of 12% was added to inhibit precipitation of the enzyme. Crystallization screens were set up using the sitting drop vapor diffusion method with an automated Phoenix (Art Robins) robot (Protein Crystallography Facility, IIT Bombay) in 96 well Intelliplate with a 1:1 mother liquor: enzyme ratio. Various commercially available screens such as PEG suite (Qiagen), PEG Rx (Qiagen), PEG Ion (Qiagen), Index and JCSG+ (Molecular Dimensions) were used for crystallization screening. The crystallization trays were incubated at 22 °C (except for TA_2799, which was incubated at 18 °C) in a vibration-free cooling incubator. The drops were regularly monitored for crystal growth under a stereomicroscope. Several conditions produced crystals, and the best crystals were grown within 51 days (Fig. S6).

### X-ray diffraction data collection and processing:

X-ray diffraction experiments were performed under liquid-nitrogen cryoconditions at 100 K. A solution of 25% (v/v) glycerol mixed with the respective mother liquor served as the optimal cryoprotectant for freezing the crystals. The crystals were briefly soaked in their corresponding cryoprotectant solutions using a nylon loop and flash frozen in a liquid nitrogen stream at 100 K prior to data collection. Data sets of TA_2799 and TA_5182 (both open and closed states) were collected using the rotation method, with 0.5° rotation per frame at a wavelength of 1.5418 Å using Cu *K*α X-ray radiation generated by a Rigaku Micromax-007HF generator equipped with R-Axis IV++ detector at the Protein Crystallography Facility, IIT Bombay. The crystal of TA_2799_L322F mutant was diffracted at the INDUS-II synchrotron beamline PX-BL-21 equipped with a MAR CCD detector, at RRCAT, Indore, India at a wavelength of 0.9789 Å. The image frames of the data set were indexed, integrated and scaled using XDS (34). The intensities were converted to structure factors using F2MTZ and CAD in CCP4 (35). The data collection and refinement statistics are summarized in Table 1.

### Structure solution and refinement

The structures of the open and the closed states of TA_5182, and the PLP bound TA_2799 and the TA_2799_L322F mutant were solved by the molecular replacement method. The monomer of the crystal structure of the ω-TA from *Pseudomonas* sp. (PDB ID: 5TI8) (61% sequence identity) was used as the initial model for solving the structure of TA_5182 in its open state. The initial phases were obtained using the MORDA pipeline of the CCP4 online server (36). For solving the structure of TA_2799, the crystal structure of the ω-TA from *P. jensenni* (PDB ID: 6G4B) (58% sequence identity) served as the initial model. The closed structure of TA_5182 was solved using the open structure TA_5182 as the initial model. The model building was done by visual inspection of electron density in COOT (37) and iterative cycles of refinement by REFMAC5 (38) or using phenix.refine of *PHENIX* (39). The covalently bound PLP was built in the *F*_*o*_*-F*_*c*_ omit electron density map and subsequently refined in the PLP bound structure of TA_2799. The solvent molecules and ions were subsequently added at peaks of electron density higher than 3σ in σ-A weighted *F*_*o*_*-F*_*c*_ electron density maps while monitoring the decrease of *R*_free_ and improvement of the overall stereochemistry and Figure of Merit (FOM). The RMSD values were calculated by superimposing the final refined structures onto their respective templates used for structure solution [TA_5182 open state (9J4Z) to 5TI8: 0.9 Å over 387 Cα atoms, TA_2799 (9J2K) to 6G4B: 0.9 Å over 451 Cα atoms].

## Data availability

The atomic coordinates of TA_2799, TA_2799_L322F, TA_5182 open and TA_5182 closed were deposited in the Protein Data Bank (PDB) with accession codes 9J2K, 9J4Y, 9J4Z and 9J50 respectively. All relevant data are available from the corresponding author upon request.

## Supporting information

This article contains supporting information (10, 11, 12, 13, 14, 15, 16, 17, 18, 22, 23, 25, 26, 40, 41, 42, 43, 44, 45, 46, 47, 48, 49, 50, 51, 52, 53, 54) (https://doi.org/10.2210/pdb3HMU/pdb, http://doi.org/10.2210/pdb6snu/pdb, http://doi.org/10.2210/pdb3gju/pdb, https://doi.org/10.2210/pdb8wqj/pdb, http://doi.org/10.2210/pdb3fcr/pdb, http://doi.org/10.2210/pdb6wnn/pdb, http://doi.org/10.2210/pdb3i5t/pdb, http://doi.org/10.2210/pdb3nui/pdb).

## Conflict of interest

The authors declare no competing interests with the contents of the article.

## Supporting information

Supporting information

Media_S1

Media_S2

Media_S3

Media_S4
