# Supplementary material for: Structural insights and rational design of Pseudomonasputida KT2440 omega transaminases for enhanced biotransformation of (R)-PAC to (1R, 2S)-Norephedrine
Source: J Biol Chem. 2025 May 26;301(7):110289. doi: 10.1016/j.jbc.2025.110289 (PMC12226076; doi:10.1016/j.jbc.2025.110289)
Supplement: Supporting information [file mmc1.pdf]

Supporting Information

Structural insights and rational design of *Pseudomonas putida* KT2440 Omega transaminases for enhanced biotransformation of (*R*)-PAC to (1*R*, 2*S*)-Norephedrine

Parijat Das[1], Santosh Noronha[2*], Prasenjit Bhaumik[1*]

[1]*Department of Biosciences and Bioengineering, Indian Institute of Technology Bombay, Powai, Mumbai-400076, India,*

[2]*Department of Chemical Engineering, Indian Institute of Technology Bombay, Powai, Mumbai-400076, India*

Running title: Rational design of omega transaminases

[*]To whom correspondence should be addressed:

Santosh Noronha, Department of Chemical Engineering, Indian Institute of Technology Bombay, Powai, Mumbai-400076, India; E-mail: noronha@iitb.ac.in

Prasenjit Bhaumik, Department of Biosciences and Bioengineering, Indian Institute of Technology Bombay, Powai, Mumbai-400076, India; E-mail: pbhaumik@iitb.ac.in

```
TA_2799         ------MSTHSSTVQNDLAALIHPNTNLAQHREVGPLV-IARGDGVRVFDEQGNAYIEAM    53
TA_5182         --MSVNNPQTREWQTLSGEHHLAPFSDYKQLKEKGPRI-ITKAQGVHLWDSEGHKILDGM    57
PsTA_5TI8       -MTKQTSAQTQHWQALSREHHLAPFTDYKQLNEKGARI-ITKAEGVYLWDSEGNKILDGM    58
HeTA_6GWI       -------MQTQDYQALDRAHHLHPFTDFKALGEEGSRV-VTHAEGVYIHDSEGNRILDGM    52
RpTA_3HMU       MATITNHMPTAELQALDAAHHLHPFSANNALGEEGTRV-ITRARGVWLNDSEGEEILDAM    59
CvTA_6S4G       ---MQKQRTTSQWRELDAAHHLHPFTDTASLNQAGARV-MTRGEGVYLWDSEGNKIIDGM    56
VpTA_6FYQ       ---------MRSLTELDKKHFIHPTSSIQEQQHKGAKVIMKEGDGIYLTDVTGKTYIDGV    51
VfTA_5ZTX       ------MNKPQSWEARAETYSLYGFTDMPSLHQRGTVV-VTHGEGPYIVDVNGRRYLDAN    53
PjTA_6TB1       -----MNQSVSSLAEKDIQYQLHPYTNARLHQELGPLI-IERGQGIYVYDDQGKGYIEAM    54
BaTA_5GHG       -----MTAQPNSLEARDIRYHLHSYTDAVRLEAEGPLV-IERGDGIYVEDVSGKRYIEAM    54
PfTA_6S54       ----MNSNNKAWLKEHNTVHMMHPMQDPKALHEQRPLI-IQSGKGVHITDVDGRRFIDCQ    55
                                     :               : :  . *  : *  *.  ::

TA_2799         SGLWSAALGFSEQRLVDAAVEQFKQLPYYHSFSHKTNAPAAALAAKLAALAP-GDLNHVF    112
TA_5182         AGLWCVAVGYGREELVQAAEKQMRELPYYNLFFQTAHPPALELAKAITDVAP-KGMTHVF    116
PsTA_5TI8       AGLWCMNVGYGRKELAEVAYKQMLELPYYNLFFQTAHPPALELAKAIADIAP-EGMNHVF    117
HeTA_6GWI       AGLWCVNLGYGRRELVEAATAQLEQLPYYNTFFKTTHPPAVRLAEKLCDLAP-AHINRVF    111
RpTA_3HMU       AGLWCVNIGYGRDELAEVAARQMRELPYYNTFFKTTHVPAIALAQKLAELAP-GDLNHVF    118
CvTA_6S4G       AGLWCVNVGYGRKDFAEAARRQMEELPFYNTFFKTTHPAVVELSSLLAEVTP-AGFDRVF    115
VpTA_6FYQ       SSLWNVNVGHGRVELAEAAAQQMKKMAFSSAFSTFSHEPAIRLAEKIASITP-EGLNAVF    110
VfTA_5ZTX       SGLWNMVAGFDHKGLIDAAKAQYERFPGYHAFFGRMSDQTVMLSEKLVEVSP-FDSGRVF    112
PjTA_6TB1       AGLWSVALGFSNQRLIKAAEQQFNTLPFYHLFNHKSHRPSIELAEKLIEMAP-VPMSKVF    113
BaTA_5GHG       SGLWSVGVGFSEPRLAEAAARQMKKLPFYHTFSYRSHGPVIDLAEKLVSMAP-VPMSKAY    113
PfTA_6S54       GGLWCVNAGYGRREIIDAVTRQMEELAYYSLFPGSTNAPAIALSQKLTEVAAEEGMVKAS    115
                ..**    *...  : ...  *   :     *          *:  :  ::       .

TA_2799         FTNSGSEANDSVVKMVWYVNNALGRPAKKKFISRQQAYHGATVAAASLTGIPSMHRDFDL    172
TA_5182         FTGSGSEGNDTVLRMVRHYWALKGKPHKQTIIGRINGYHGSTFAGACLGGMSGMHEQGGL    176
PsTA_5TI8       FTGSGSESNDTVLRMVRHYWSIKGKPQKKVVIGRWNGYHGSTVAGVSLGGMKALHSQGDL    177
HeTA_6GWI       FTGSGSEANDTVLRMVRRYWALKGQPDKQWIIGRENAYHGSTLAGMSLGGMAPMHAQGGP    171
RpTA_3HMU       FAGGGSEANDTNIRMVRTYWQNKGQPEKTVIISRKNAYHGSTVASSALGGMAGMHAQSGL    178
CvTA_6S4G       YTNSGSESVDTMIRMVRRYWDVQGKPEKKTLIGRWNGYHGSTIGGASLGGMKYMHEQGDL    175
VpTA_6FYQ       FTSGGSESNDSAVKLVRHYWKIQGKPNKRKIISLKRSYHGVAAASTSVTGIPEFWGMAGH    170
VfTA_5ZTX       YTNSGSEANDTMVKMLWFLHAAEGKPQKRKILTRWNAYHGVTAVSASMTGKPYN-SVFGL    171
PjTA_6TB1       FTNSGSEANDTVVKFVWYLNNALGKPAKKKFISRVNGYHGVTVASASLTGLPGNQRGFDL    173
BaTA_5GHG       FTNSGSEANDTVVKLIWYRSNALGEPERKKIISRKRGYHGVTIASASLTGLPNNHRSFDL    173
PfTA_6S54       FGLGGSDAVETALKIARQYWKLEGQPDKVKFVSLYNGYHGLNFGGMSACGGNAWKSSYEP    175
                :  .**:. :: :::        *.* :  .:   ..***     . .  *

TA_2799         PAIPVHHLTCPNFYRFARPGESQEAFTVRLANELERYILAEGPETIAAFIGEPVIAAGGV    232
TA_5182         PIPGIVHIPQPYWFGEGGD-MTPDEFGVWAAEQLEKKILEVGEDNVAAFIAEPIQGAGGV    235
PsTA_5TI8       PIPGIVHIAQPYWYGEGGD-MSAEEFGVWAAEQLEKKILEVGEENVAAFIAEPIQGAGGV    236
HeTA_6GWI       CVPGIAHIRQPYWFGEGRD-MSPEAFGQTCAEALEEKILELGEEKVAAFIAEPVQGAGGA    230
RpTA_3HMU       -IPDVHHINQPNWWAEGGD-MDPEEFGLARARELEEAILELGENRVAAFIAEPVQGAGGV    236
CvTA_6S4G       PIPGMAHIEQPWWYKHGKD-MTPDEFGVVAARWLEEKILEIGADKVAAFVGEPIQGAGGV    234
VpTA_6FYQ       MMTDFLHVDTHYNNTT-----------EQAVQSLCQAIEEAGPETIAAFFAEPVQGAGGV    219
VfTA_5ZTX       PLPGFVHLTCPHYWRYGEEGETEEQFVARLARELEETIQREGADTIAGFFAEPVMGAGGV    231
PjTA_6TB1       PLPGFLHVGCPHHYRFALAGESEEHFADRLAVELEQKILAEGPETIAAFIGEPLMGAGGV    233
BaTA_5GHG       PIDRILHTGCPHYYREGQAGETEEQFATRLADELEQLIIAEGPHTIAAFIGEPVMGAGGV    233
PfTA_6S54       LMPGFFQVESPHLYRNPFT-NDPEELAEICAQILERQIEMQAPGTVAALIAEPIQGAGGV    234
                    . :                       .  * . *   .   :*.:..**: .***.

TA_2799         IPPPTGYWAAIQAVCKRYDILVVIDEIITGFGRLGTMFGSQLYGIQPDIMVLSKQLTSSY    292
TA_5182         IIPPETYWPKVKEILARYDILFVADEVICGFGRTGEWFGSDYYDLKPDLMTIAKGLTSGY    295
PsTA_5TI8       IVPPDTYWPKIREILAKYEILFIADEVICGFGRTGEWFGSQYYGNAPDLMPIAKGLTSGY    296
HeTA_6GWI       IMPPESYWPAVKKVLAKYDILLVADEVICGFGRLGEWFGSQHYGLEPDLMPIAKGLSSGY    290
RpTA_3HMU       IVAPDSYWPEIQRICDKYDILLIADEVICGFGRTGNWFGTQTMGIRPHIMTIAKGLSSGY    296
CvTA_6S4G       IVPPATYWPEIERICRKYDVLLVADEVICGFGRTGEWFGHQHFGFQPDLFTAAKGLSSGY    294
VpTA_6FYQ       IIPPEDYFLRIREVCNAYGILFVADEVITGFGRTGKMFGIENWDVIPDVMTFAKGVTSGY    279
VfTA_5ZTX       IPPAKGYFQAILPILRKYDIPVISDEVICGFGRTGNTWGCVTYDFTPDAIISSKNLTAGF    291
PjTA_6TB1       IVPPRTYWEKIQKVCRKYDILVIADEVICGFGRTGQMFGSQTFGIQPDIMVLSKQLSSSY    293
BaTA_5GHG       VVPPKTYWEKVQAVLKRYDILLIADEVICGFGRTGNLFGSQTFDMKPDILVMSKQLSSSY    293
PfTA_6S54       IVPPASYWPRLRQICDKYDILLIADEVITGLGRSGSLFGSRGWGVKPDIMCLAKGISSGY    294
                :     *:  :  :   * : .: **:* *:** *  :*    .  *. :  :* :::.:
```

```
TA_2799         QPLAAVVVSDAMNDVLVSQSQRLGAFAHGLTCTGHPVATAVALENIRIIEERDLVGHVQH    352
TA_5182         IPMGGVIVRDTVAKV-IS---EGGDFNHGFTYSGHPVAAAVGLENLRILRDEKIVEKART    351
PsTA_5TI8       IPMGGVIVRDEIVDT-LN---EGGEFYHGFTYSGHPVAAAVALENIRILREEKIVETVKA    352
HeTA_6GWI       LPIGGVLVGDRVAETLIE---EGGEFFHGFTYSGHPTCAAVALKNLELLEAEGVVDRVRD    347
RpTA_3HMU       APIGGSIVCDEVAHVI-----GKDEFNHGYTYSGHPVAAAVALENLRILEEENILDHVRN    351
CvTA_6S4G       LPIGAVFVGKRVAEGLI----AGGDFNHGFTYSGHPVCAAVAHANVAALRDEGIVQRVKD    350
VpTA_6FYQ       FPLGGVVVSDPIHEVLKE--KSVGTLFHGFTYSGHPTAAAVALKNIAIIKEERLVENSKR    337
VfTA_5ZTX       FPMGAVILGPELSKRLETAIEAIEEFPHGFTASGHPVGCAIALKAIDVVMNEGLAENVRR    351
PjTA_6TB1       QPIAAILINAPVFEGIADQSQALGALGHGFTGSGHPVATAVALENLKIIEEESLVEHAAQ    353
BaTA_5GHG       LPISAFLINERVYAPIAEESHKIGTLGTGFTASGHPVAAAVALENLAIIEERDLVANARD    353
PfTA_6S54       VPLSATLVNSRVARAWERDAGFTSVYMHGYTYSGHPVSCAAALAAIDIVLQENLAENARV    354
                 *:.. .:   :                 * * :***. * .   :  :  . :

TA_2799         -LAPVFQRHLRAFE-DHPLVGNVRGVGLMGGIELVADKATRQPFAQ-PGTLGGYVFKQAH    409
TA_5182         EAAPYLQKRLRELQ-DHPLVGEVRGLGMLGAIELVKDKATRSRYEG-K-GVGMICRTFCF    408
PsTA_5TI8       ETAPYLQKRWQELA-DHPLVGEARGVGMVGALELVKNKKTRERFE--N-GVGMLCREHCF    408
HeTA_6GWI       DLGPYLAERWASLV-DHPIVGEARSLGLMGALELVADKTTGQRFDKSL-GAGNLCRDLCF    405
RpTA_3HMU       VAAPYLKEKWEALT-DHPLVGEAKIVGMMASIALTPNKASRAKFASEPGTIGYICRERCF    410
CvTA_6S4G       DIGPYMQKRWRETFSRFEHVDDVRGVGMVQAFTLVKNKAKRELFPD-FGEIGTLCRDIFF    409
VpTA_6FYQ       -MGDALLHGLKKVKNRLEIVGDVRFVGLLGAVELMQNPATNKPFSS-NLQVAPKVIEALH    395
VfTA_5ZTX       -LAPRFEERLKHIA-ERPNIGEYRGIGFMWALEAVKDKASKTPFDG-NLSVSERIANTCT    408
PjTA_6TB1       -MGQLLRSGLQHFI-DHPLVGEIRGCGLIAAVELVGDRVSKAPYQA-LGTLGRYMAGRAQ    410
BaTA_5GHG       -RGTYMQKRLRELQ-DHPLVGEVRGVGLIAGVELVTDKQAKTGLEP-TGALGAKANAVLQ    410
PfTA_6S54       -VGDYFLEKLLILKDKHRAIGDVRGKGLMLAVELVKERATKEPFGP-ADAYPLAISEACV    412
                  .  :             :.: :  *:: ..    :

TA_2799         KHGLIIRAIY----DTIAFCPPLITTQDDIEAIFSAFERTLADATDWARSQHLL------    459
TA_5182         ENGLIMRAVG----DTMIIAPPLVISHAEIDELVEKARKCLDLTLEAIQ-----------    453
PsTA_5TI8       RNGLIMRAVG----DTMIISPPLVITKPEIDELITLARKCLDQTAAVALS----------    454
HeTA_6GWI       ANGLVMRSVG----DTMIISPPLVIRREEIDELVELARRALDETARQLTQVPHTQEEPTA    461
RpTA_3HMU       ANNLIMRHVG----DRMIISPPLVITPAEIDEMFVRIRKSLDEAQAEIEKQGLMKSAA--    464
CvTA_6S4G       RNNLIMRACG----DHIVSAPPLVMTRAEVDEMLAVAERCLEEFEQTLKARGLA------    459
VpTA_6FYQ       ELGVICRSVTYDHTNIICLAPPLIINQKQVDKLVEVIYEAILKVQQQLGIKAE-------    448
VfTA_5ZTX       DLGLICRPLG----QSVVLCPPFILTEAQMDEMFDKLEKALDKVFAEVA-----------    453
PjTA_6TB1       EHGMITRAMG----DAVAFCPPLIVNEQEVGMIVERFARALDDTTQWVGPGG--------    458
BaTA_5GHG       ERGVISRAMG----DTLAFCPPLIINDQQVDTMVSALEATLNDVQASLTR----------    456
PfTA_6S54       NNGVMIRTIV----NKLIISPPLTFTTEHVDEVIEVLDRAFVANPW--------------    454
                  .:: *       : :  .**:     .:  :.      :
```

Supporting data 1: Multiple sequence alignment of TA enzymes from different organisms showing the sequence conservation among fold type I omega transaminases available in the PDB. The residues of interest are highlighted in yellow and marked with a red arrow. The N115 in TA_2799 is somewhat conserved in other TAs, but substituted by a glycine in a few including TA_5182. On the other hand, a conserved aromatic amino acid (phenylalanine/tyrosine) can be found in other TAs at the L322 position of TA_2799. Abbreviations: PsTA: Pseudomonas TA; HeTA: *Halomonas elongata* TA; RpTA: *Ruegeria pomeroyi* TA; CvTA: *Chromobacterium violaceum* TA; VpTA: *Virgibacillus pantothenticus* TA; VfTA: *Vibrio fluvialis* TA; PjTA: *Pseudomonas jensenni* TA; BaTA: *Brucella anthropi* TA; PfTA: *Pseudomonas fluorescens* TA. The four-letter codes following the abbreviations correspond to the PDB IDs. The sequence alignment was performed using Clustal Omega web server (https://www.ebi.ac.uk/jdispatcher/msa/clustalo).

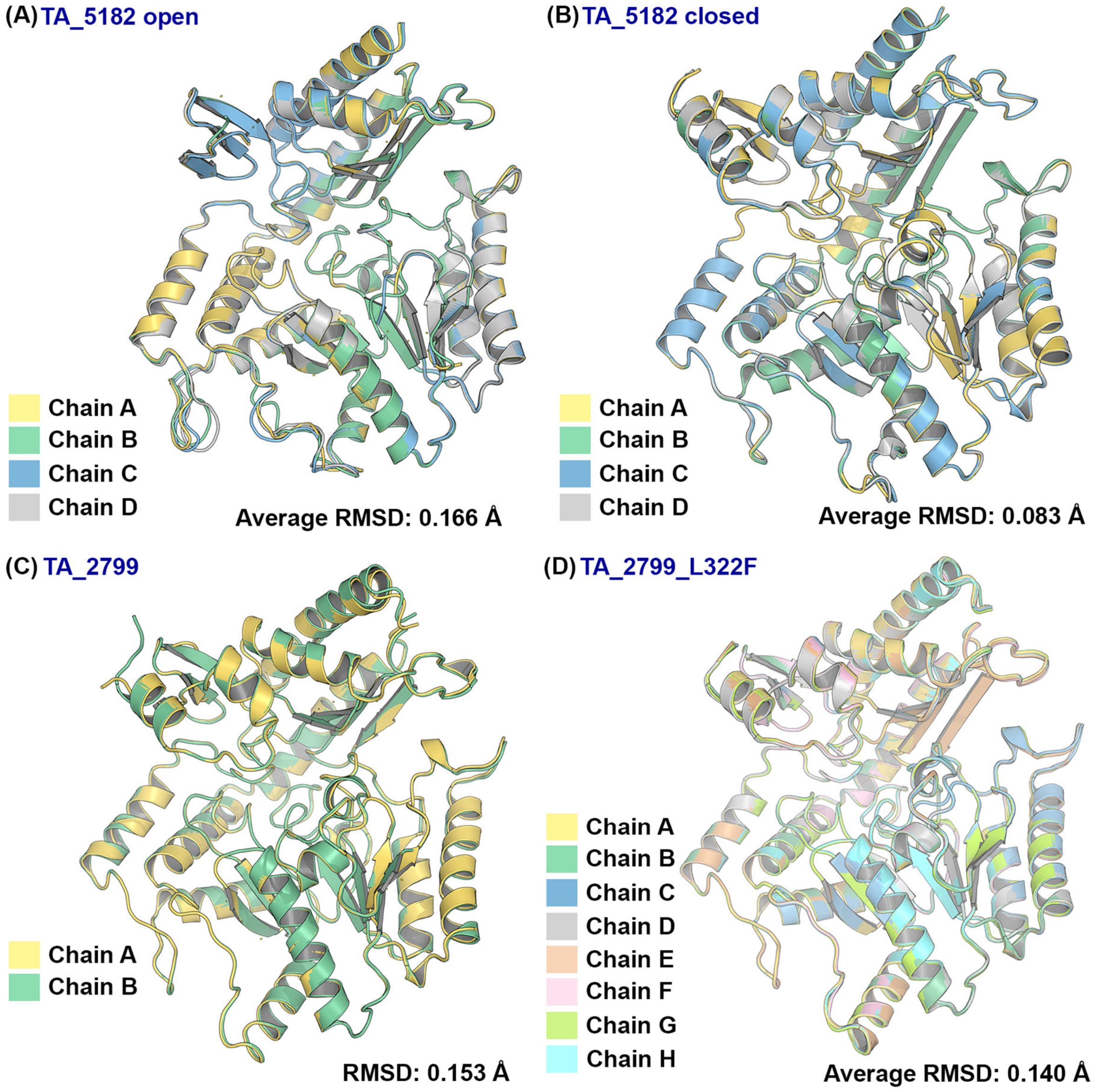

Figure S1: Superimposition of each subunit on Chain A of each enzyme. **(A)** TA_5182 open state. **(B)** TA_5182 closed state. **(C)** TA_2799 and **(D)** TA_2799_L322F. The average RMSD values of the superimpositions are mentioned in Å. Each chain is colored differently. Except for TA_5182 open state, all the subunits are almost identical to each other. In the open state of TA_5182, the major differences can be seen at the outer and roof loop regions. A comprehensive list of the individual RMSD values is calculated in Table S1.

Table S1: RMSD values (Å) of each monomer superposed on Chain A of each enzyme

	Chain B	Chain C	Chain D	Chain E	Chain F	Chain G	Chain H
**TA_5182 open**	0.140 (344)	0.179 (341)	0.180 (329)				
**TA_5182 closed**	0.081 (380)	0.086 (384)	0.083 (390)				
**TA_2799**	0.153 (372)						
**TA_2799_L322F**	0.183 (413)	0.107 (397)	0.177 (380)	0.140 (409)	0.139 (387)	0.114 (415)	0.124 (402)

Values in brackets indicate the number of Cα atoms used for the alignment

Table S2: Overall comparison of the TA_5182 open state with known structures in PDB using DALI.

PDB	Organism	Ref.	Open state of TA_5182									
			Chain A to Chain A					Chain A to Chain B				
			Z-Score	RMSD(Ă)	Lali	Nres	% Identity	Z-Score	RMSD(Ă)	Lali	Nres	% Identity
5ti8	*Pseudomonas sp.* M1	(1)	61.4	0.9	387	387	78	60.7	0.9	384	384	78
6gwi	*Halomonas elongata*	(2)	57.4	1.8	399	449	62	53.7	1.9	400	450	62
7qx3	*Acidihalobacter*	(3)	57.3	1.4	402	422	64	56.5	1.4	396	418	63
7qyf	*Acidihalobacter*	(3)	57.2	1.4	398	418	64	56.2	1.4	400	424	64
7qx0	*Acidihalobacter*	(3)	55.8	1.8	398	420	63	56.6	1.5	397	443	64
4a6u	*Chromobacterium violaceum*	(4)	55.1	1.4	379	389	56	55.1	1.4	379	389	56
3hmu	*Ruegeria pomeroyi*	(5)	55	1.8	398	464	57	55.6	1.8	398	460	56
4a6r	*Chromobacterium violaceum*	(4)	54.8	1.4	396	423	55	54.7	1.4	396	422	55
7ypm	*Caulobacter sp.* D5	(6)	54.3	1.9	400	455	53	54.1	2.0	400	456	53
4ba4	*Chromobacterium violaceum*	(7)	54.3	1.4	396	427	55	54.4	1.4	397	427	55
6snu	*Chromobacterium violaceum*	(8)	54.2	1.9	398	452	54	54.1	1.9	398	453	54
4a6t	*Chromobacterium violaceum*	(4)	54.2	1.9	399	454	54	54.1	1.9	398	454	54
4ah3	*Chromobacterium violaceum*	(7)	54.2	1.9	398	455	54	54	1.9	398	455	54
7ypn	*Caulobacter sp* D5	(6)	54.1	2.0	400	454	52	54.1	2.0	400	454	52
6s4g	*Chromobacterium violaceum*	(9)	54.1	1.9	398	453	54	54	1.9	398	453	54
4ba5	*Chromobacterium violaceum*	(7)	53.5	1.5	396	427	55	53.8	1.5	398	427	54
5ghf	*Brucella anthropi*	(10)	53.2	1.6	383	431	43	51.9	2.0	398	424	42
5kr3	*Pseudomonas sp.*	(11)	51.8	1.9	393	458	43	51.6	1.9	394	458	43
3gju	*Mesorhizobium loti*	(12)	51.7	2.1	395	458	40			NA		
7b4j	*Pseudomonas jensenni* 6 fold mutant	(13)	51.6	2.1	399	449	41	51.1	2.1	399	452	41
6g4b	*Pseudomonas jensenni*	(14)	51.6	2.1	399	451	41	48.5	2.1	399	453	41
5kqw	*Pseudomonas sp.*	(11)	51.6	2.1	398	457	44	51.6	2.1	398	458	44
5kr4	*Pseudomonas sp.*	(11)	51.6	1.9	395	457	42	51.6	1.9	395	457	42
5ghg	*Brucella anthropi*	(10)	51.5	2.0	398	425	41	53	1.6	385	433	43
5kr6	*Pseudomonas sp.*	(11)	51.5	2.0	397	457	45	51.9	1.9	394	460	45
5kqt	*Pseudomonas sp.*	(11)	51.5	2.1	397	457	42			NA		
6g4d	*Pseudomonas jensenni*	(14)	51.4	2.1	399	451	41	51.1	2.1	399	453	41
6tb0	*Pseudomonas jensenni* 4 fold mutant	(15)	51.3	2.1	399	451	41	48.3	2.1	399	453	41
6g4e	*Pseudomonas jensenni*	(14)	51.3	2.1	399	451	41	50.7	2.1	398	452	41
6g4f	*Pseudomonas jensenni*	(14)	51.3	2.1	399	451	41	48.4	2.1	399	453	41
5kr5	*Pseudomonas sp.*	(11)	51.3	1.9	392	454	44	48.2	1.9	393	455	44

Table S2 (continued)

PDB	Organism	Ref.	Open state of TA_5182									
			Chain A to Chain A					Chain A to Chain B				
			Z-Score	RMSD(Ǎ)	Lali	Nres	% Identity	Z-Score	RMSD(Ǎ)	Lali	Nres	% Identity
5kqu	*Pseudomonas sp.*	(11)	51.2	1.9	394	457	43	48.2	1.9	395	458	43
6io1	*Thermomicrobium roseum*	(16)	51	2.2	392	447	42	50.6	2.2	392	448	41
8wqj	*Shimia marina*	(17)	50.8	1.8	398	472	57	55.8	1.8	398	462	57
3fcr	*Ruegeria sp.* TM1040	(18)	50.5	2.0	392	458	41			NA		
6wnn	*Bacillus subtilis*	(19)	50.4	2.2	386	421	33	49.6	2.2	382	416	33
6tb1	*Pseudomonas jensenni* 4 fold mutant	(15)	50.4	2.1	399	451	41	47.5	2.1	399	453	41
3i5t	*Cereibacter sphaeroides* 2.4.1	(20)	50.3	2.1	393	444	37	50.1	2.1	392	442	37
6fyq	*Virgibacillus pantothenticus*	(21)	50.3	2.1	389	443	40			NA		
3dod	*Bacillus subtilis*	(22)	49.7	2.1	381	417	32	49.5	2.1	380	416	32
3drd	*Bacillus subtilis*	(22)	49.6	2.1	381	419	32	49.4	2.1	380	417	32
5ztx	*Vibrio fluvalis* JS17	(23)	49.4	1.8	392	424	36	49.5	2.0	395	435	37
3nui	*Vibrio fluvalis* JS17	(24)	49.4	1.9	394	425	36	49.4	2.0	395	419	37
4e3r	*Vibrio fluvalis*	(25)	49.1	2.1	395	452	35	46.3	2.3	399	452	34
6g4c	*Pseudomonas jensenni*	(14)	48.8	2.1	399	452	41	48.5	2.1	399	453	41
6s54	*Pseudomonas fluoroscens*	(26)	48.4	2.4	395	453	38	48.3	2.3	395	450	38
5lh9	*Pseudomonas sp.*	(27)	48.4	2.4	395	444	34	45.5	2.4	395	447	33
3du4	*Bacillus subtilis*	(22)	48.1	2.2	392	449	32	45.6	2.2	393	449	32
5lha	*Pseudomonas sp.*	(27)	48.1	2.4	395	447	34	47.7	2.5	395	446	34
4e3q	*Vibrio fluvalis*	(25)	47.8	2.3	399	452	35	49.1	2.3	399	450	35
4grx	*Paranitrococcus denitrificans*	(28)	46.2	2.4	399	449	35	49.1	2.3	399	449	35
4uhn	*Pseudomonas sp.*	(29)	46.2	2.3	380	435	35			NA		
4uho	*Pseudomonas sp.*	(29)	46.2	2.3	380	435	35			NA		
4uhm	*Pseudomonas sp.*	(29)	46.1	2.3	380	435	35			NA		
6jix	*Bifidobacterium catenulatum* PV20-2	(30)	45.8	2.2	387	448	30	44.5	2.3	390	448	30
4bq0	*Pseudomonas aeruginosa* PAO1	(31)	45.7	2.3	381	439	36	46.1	2.3	381	436	36
4b9b	*Chromobacterium violaceum*	(7)	45.6	2.4	381	436	36	45.8	2.3	381	435	36
4b98	*Chromobacterium violaceum*	(7)	45.6	2.3	380	441	36	45.6	2.3	380	440	36
6zhk	*Methanocaldococcus jannaschii* DSM 2661	(32)	44.7	2.6	381	438	30	44.6	2.6	381	438	30
7qzj	*Streptomyces sp.* (HPH0547)	(33)	43.9	2.5	375	422	29	46.5	2.4	375	422	29

Lali: Local Alignment length index **Nres: Number of residues**

Table S3: Overall comparison of the PLP bound TA_2799 with known structures in PDB using DALI.

PDB	Organism	Ref.	PLP bound TA_2799									
			Chain A to Chain A					Chain A to Chain B				
			Z-Score	RMSD(Å)	Lali	Nres	% Identity	Z-Score	RMSD(Å)	Lali	Nres	% Identity
6g4e	*Pseudomonas jensenni*	(14)	62.7	0.9	451	451	58	61.7	1	449	452	58
6tb0	*Pseudomonas jensenni* 4 fold mutant	(15)	62.6	0.9	451	451	58	59	1	450	453	58
6g4d	*Pseudomonas jensenni*	(14)	62.6	0.9	451	451	58	62	1	450	453	58
6g4b	*Pseudomonas jensenni*	(14)	62.6	0.9	451	451	58	59	1	450	453	58
7b4j	*Pseudomonas jensenni* 6 fold mutant	(13)	62.5	0.8	449	449	57	61.8	0.9	449	452	57
6g4f	*Pseudomonas jensenni*	(14)	62.4	0.9	451	451	58	59	1	450	453	58
6tb1	*Pseudomonas jensenni* 4 fold mutant	(15)	61.7	0.9	451	451	58	58.1	1	450	453	58
5ghg	*Brucella anthropi*	(10)	60.8	1.1	425	425	53	60.1	1.2	433	433	52
5ghf	*Brucella anthropi*	(10)	60.2	1.2	431	431	52	61.3	1.1	424	424	53
6g4c	*Pseudomonas jensenni*	(14)	59.7	0.9	452	452	58	59.1	1	451	453	58
5kr6	*Pseudomonas sp.*	(11)	59.4	1.3	450	457	44	59.4	1.3	450	460	44
5kqw	*Pseudomonas sp.*	(11)	59.2	1.3	451	457	42	59.2	1.3	451	458	42
5kr4	*Pseudomonas sp.*	(11)	59	1.3	451	457	41	58.9	1.3	450	457	41
5kr3	*Pseudomonas sp.*	(11)	58.9	1.3	449	458	42	59.1	1.3	451	458	42
3gju	*Mesorhizobium loti*	(12)	58.8	1.5	449	458	41			NA		
5kqu	*Pseudomonas sp.*	(11)	58.7	1.3	451	457	40	55.9	1.4	451	458	40
5kqt	*Pseudomonas sp.*	(11)	58.7	1.3	450	457	41			NA		
4e3r	*Vibrio fluvalis*	(25)	58	1.6	451	452	37	54.7	1.6	451	452	37
7ypn	*Caulobacter sp* D5	(6)	57.9	1.5	450	454	41	57.8	1.5	451	455	41
6io1	*Thermomicrobium roseum*	(16)	57.9	1.4	443	447	42	58	1.4	445	448	42
7ypm	*Caulobacter sp.* D5	(6)	57.8	1.5	450	455	42	57.7	1.5	450	456	42
3fcr	*Ruegeria sp.* TM1040	(18)	57.8	1.4	449	458	37			NA		
6snu	*Chromobacterium violaceum*	(8)	57.6	1.4	449	452	37	57.5	1.4	450	453	37
6s4g	*Chromobacterium violaceum*	(9)	57.5	1.4	451	453	37	57.3	1.4	451	453	37
4a6t	*Chromobacterium violaceum*	(4)	57.4	1.4	451	454	37	57.3	1.4	451	454	37
4ah3	*Chromobacterium violaceum*	(7)	57.4	1.4	452	455	37	57.3	1.4	452	455	37
6gwi	*Halomonas elongata*	(2)	57.3	1.5	447	449	41	54.3	1.5	447	450	41
3hmu	*Ruegeria pomeroyi*	(5)	57.2	1.4	450	464	42	58	1.3	450	460	42
5ztx	*Vibrio fluvalis* JS17	(23)	56.6	1.5	423	424	38	50.1	1.9	427	435	36
3nui	*Vibrio fluvalis* JS17	(24)	56.5	1.5	423	425	38	50.2	1.7	408	419	37
4e3q	*Vibrio fluvalis*	(25)	56.4	1.6	451	452	37	57.9	1.5	450	450	37

Table S3 (continued)

PDB	Organism	Ref.	PLP bound TA_2799									
			Chain A to Chain A					Chain A to Chain B				
			Z-Score	RMSD(Å)	Lali	Nres	% Identity	Z-Score	RMSD(Å)	Lali	Nres	% Identity
7qx0	*Acidihalobacter*	(3)	56.1	1.7	418	420	41	54.4	1.8	437	443	39
3i5t	*Cereibacter sphaeroides* 2.4.1	(20)	56.1	1.6	440	444	35	56	1.7	440	442	35
5kr5	*Pseudomonas sp.*	(11)	55.7	1.3	449	455	42	59.1	1.3	448	454	42
6fyq	*Virgibacillus pantothenticus*	(21)	55.1	1.5	436	443	38			NA		
4grx	*Paranitrococcus denitrificans*	(28)	54.9	1.4	449	449	37	58	1.4	449	449	37
5lh9	*Pseudomonas sp.*	(27)	54.7	1.6	436	444	34	51.9	1.7	439	447	33
5lha	*Pseudomonas sp.*	(27)	54.7	1.7	440	447	33	54.6	1.7	439	446	33
7qyf	*Acidihalobacter*	(3)	54.5	1.7	415	418	40	53.5	2	420	424	41
6s54	*Pseudomonas fluoroscens*	(26)	54.3	1.7	445	453	32	54.3	1.7	445	450	32
7qx3	*Acidihalobacter*	(3)	54.2	1.9	418	422	40	54	1.7	411	418	40
3du4	*Bacillus subtilis*	(22)	54	1.6	445	449	30	51.8	1.6	444	449	30
4ba4	*Chromobacterium violaceum*	(7)	53.8	1.8	420	427	37	53.7	1.8	422	427	37
4a6r	*Chromobacterium violaceum*	(4)	53.8	1.8	416	423	38	53.7	1.8	415	422	37
8wqj	*Shimia marina*	(17)	53.5	1.4	451	472	43	58.3	1.3	451	462	43
4ba5	*Chromobacterium violaceum*	(7)	53.2	1.8	419	427	37	54	1.9	422	427	36
7qzj	*Streptomyces sp.* (HPH0547)	(33)	52.1	1.7	417	422	30	49.5	1.7	418	422	30
6hx9	*Pseudomonas putida* KT2440	(34)	51.8	1.7	381	384	40	51.8	1.6	379	383	40
5ti8	*Pseudomonas sp* M1	(1)	51.6	1.7	383	387	43	51.3	1.8	380	384	43
3dod	*Bacillus subtilis*	(22)	51.1	1.5	416	417	30	50.9	1.6	415	416	30
6wnn	*Bacillus subtilis*	(19)	51.1	1.5	416	421	30	50.7	1.5	412	416	30
4a6u	*Chromobacterium violaceum*	(4)	50.9	1.7	386	389	36	50.4	1.8	386	389	37
3drd	*Bacillus subtilis*	(22)	50.8	1.5	415	419	30	50.8	1.5	413	417	30
4uhn	*Pseudomonas sp.*	(29)	50.8	1.7	425	435	32			NA		
4uho	*Pseudomonas sp.*	(29)	50.8	1.7	425	435	32			NA		
4uhm	*Pseudomonas sp.*	(29)	50.7	1.7	425	435	32			NA		
6jix	*Bifidobacterium catenulatum* PV20-2	(30)	50.5	1.6	428	448	28	49	1.6	429	448	28
6zhk	*Methanocaldococcus jannaschii* DSM 2661	(32)	50.5	1.8	429	438	31	50.5	1.8	430	438	31
4b9b	*Chromobacterium violaceum*	(7)	50.3	1.8	427	436	33	50.5	1.7	425	435	33
4bq0	*Pseudomonas aeruginosa* PAO1	(31)	50.3	1.8	430	439	33	50.7	1.8	427	436	33
4b98	*Chromobacterium violaceum*	(7)	50.1	1.9	430	441	33	50.1	1.9	430	440	33

Lali: Local Alignment length index **Nres: Number of residues**

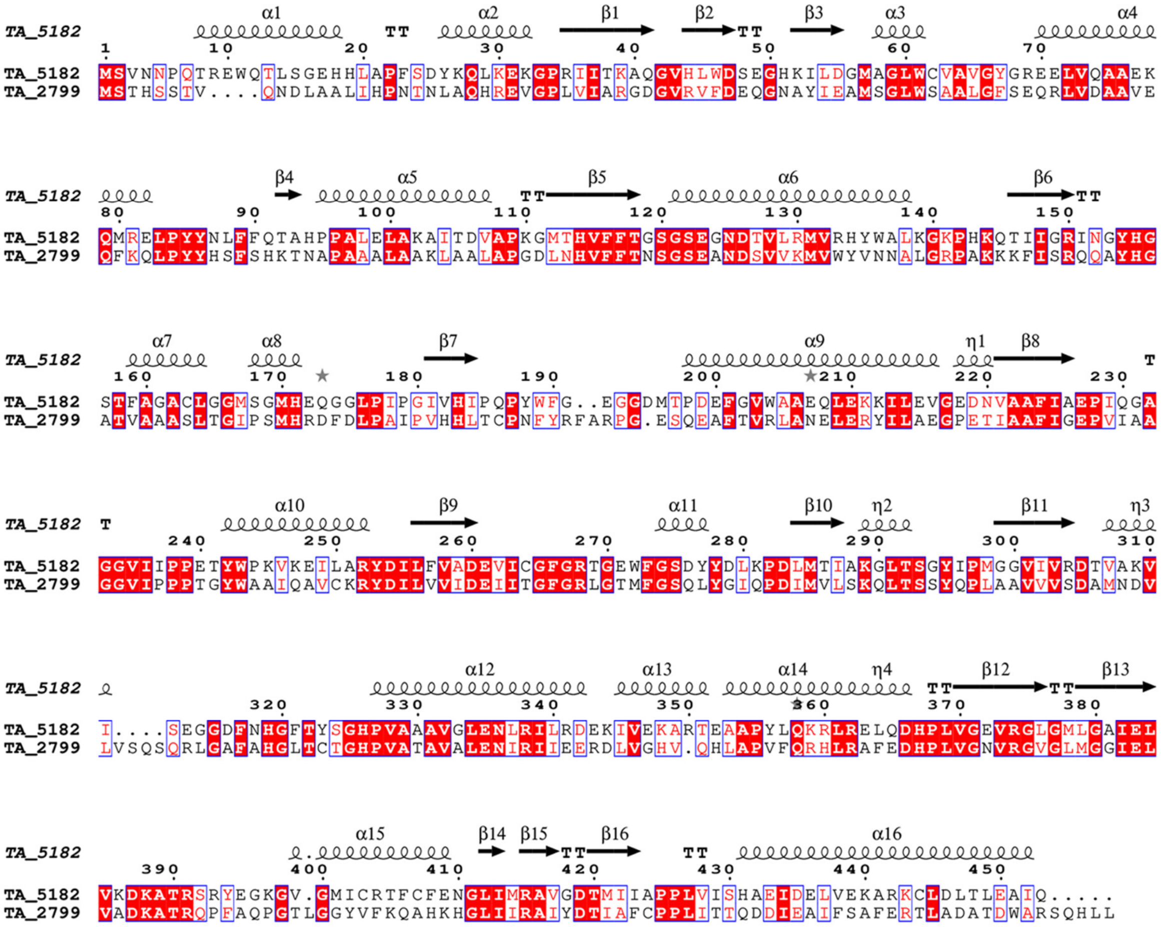

Figure S2: Sequence alignment of TA_2799 and TA_5182. Secondary structural elements of TA_5182 crystal structure is also shown on the top of the alignment. The overall sequence identity of the two enzymes in 38.49%, however the residues around the active site and the major secondary structural elements are mostly conserved (highlighted by red).

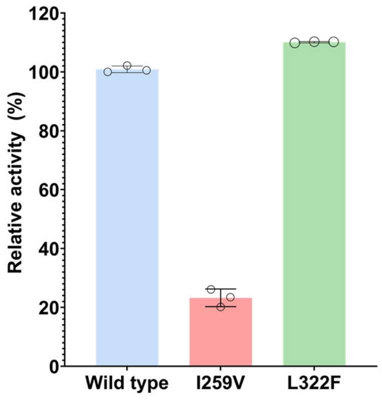

Figure S3: Relative activity of TA_2799 wild type enzyme and its mutants towards conversion of 5mM *(R)*-PAC in the presence of 10 mM *(S)*-MBA and 400 µM PLP. The I259V mutants retains only 20% of the activity, whereas the L322F mutant shows ~110% activity as compared to the wild type TA_2799 enzyme. Individual datapoints are represented by a ◦ symbol. Error bars represent the standard deviation of mean.

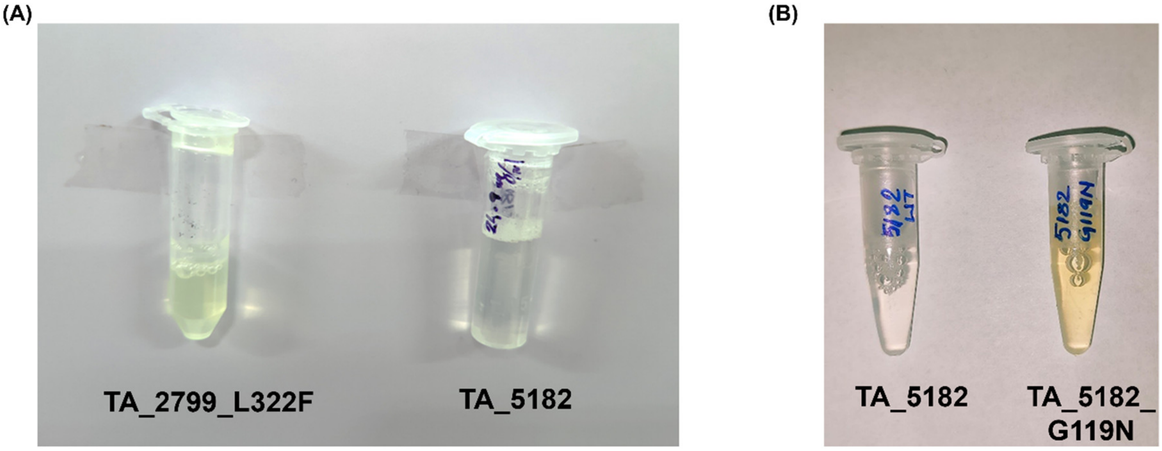

Figure S4: Purified protein showing yellow colouration in **(A)** TA_2799_L322F and **(B)** TA_5182_G119N in comparison with wild type TA_5182.

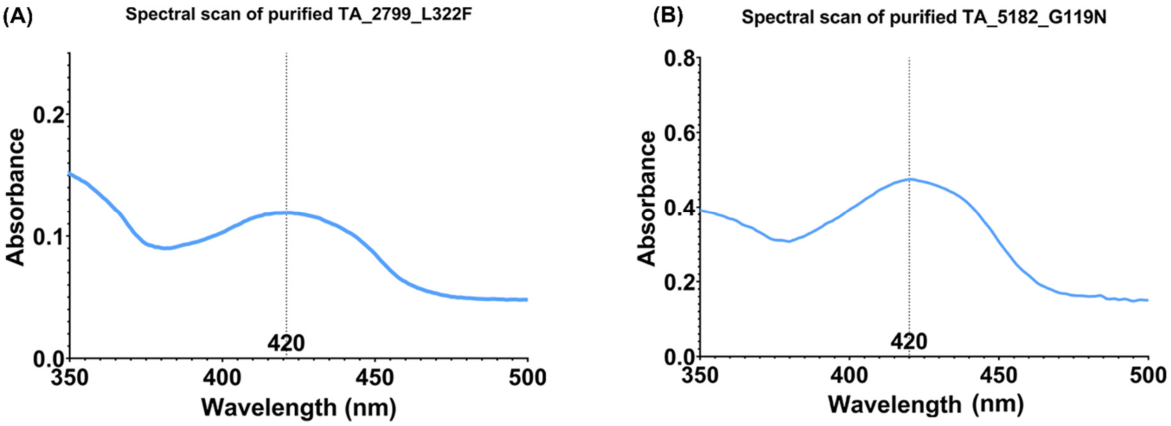

Figure S5: Spectral scan of **(A)** TA_2799_L22F and **(B)** TA_5182_G119N showing absorption peak at 420 nm corresponding to PLP bound form of the protein. The spectral scans were performed in triplicates, and one dataset has been plotted.

Supporting data 2:

Crystallization of the enzymes:

Crystallization screening drops were set in 480 different conditions in buffers with and without PLP. Both the transaminases formed crystals after at least 50 days of incubation. For TA_2799, very tiny crystals were formed in the absence of the cofactor molecule, which did not diffract. On co-crystallization of TA_2799 with 1mM PLP, a single large and robust crystal was formed in the condition 0.2 M Ammonium acetate, 0.1 M Bis-Tris 5.5, 25% w/v PEG 3,350 (INDEX G6), with a visible yellow colour signifying the crystallization of the holo enzyme (Figure S1). The crystal diffracted to a resolution of 1.67 Å at the corner of the square detector, however, the data was limited to the detector edge at 1.76 Å.

The mutant enzyme TA_2799_L322F was also crystallized in a condition containing 0.2 M lithium sulphate, 0.1 M bis-Tris pH 5.5, 25 % PEG 3350 (JCSG+ H9). Crystals were observed after 52 days of incubation at 18°C and the crystals diffracted to a resolution of 2.67 Å.

We were not able to grow good quality crystals of the wild-type TA_5182 in its PLP bound holo form. Co-crystallization with 1 mM PLP led to formation of extremely small and fragile crystals in the drops. However, TA_5182 was crystallized in two different apo forms. The open state of the enzyme crystallized in the condition containing 0.1 M Bis-Tris pH 5.5, 2 M ammonium sulphate, (INDEX A3 and JCSG+ G11) and these crystals diffracted to a resolution of 3.0 Å, but cutoff at 3.4 Å. We were also able to crystallize TA_5182 in its closed state, in the condition 1.4 M sodium potassium phosphate, pH 8.2, (Index B7). The data collection and refinement statistics are reported in Table 1.

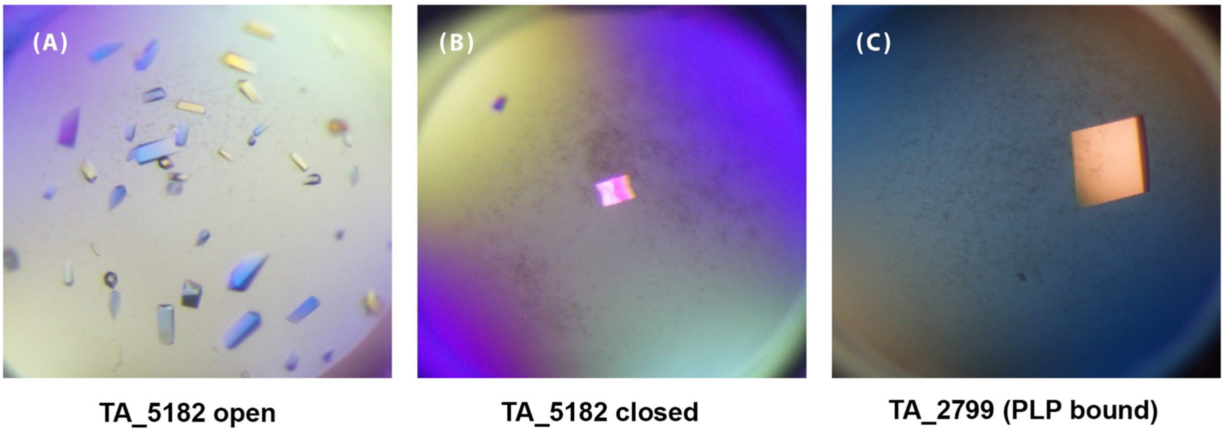

Figure S6: Crystallization drops of **(A)** TA_5182 in open state **(B)** TA_5182 in closed state and **(C)** TA_2799 in PLP bound holo state.

Supporting data 3:

Thermal shift assay:

Fluorescence based assay was used to determine the Tm_{app} values of the TA enzymes and their mutants. 100 μL of the protein solution at a final concentration of 10 mg/mL with different solvents were prepared in 50 mM HEPES buffer (pH 8.0). 22.5 μL of each of these solutions and 2.5 μL of 50x stock solution of SYPRO orange were added to Genaxy Low Profile PCR (Polymerase Chain Reaction) tubes, mixed thoroughly, and sealed with the provided caps. The assay was performed in BioRad Real Time PCR machine (Bio-Rad, USA) with a linear gradient of increasing temperature (1°C/min) from 15°C to 85°C. The temperature at the maximum rate of fluorescence change (dRFU/dT) was taken as the Tm_{app}.

Thermal stability of TA_2799 and TA_5182 and their mutants with various additives:

The wild type enzymes and TA_2799_L322F were assessed for the evaluation of their thermal stability with various additives added to the buffer. The Tm_{app} of wild-type TA_5182 was found to be 57.5°C, while for wild type TA_2799 it was 56.6°C. Addition of glycerol and sucrose did not have a significant effect on the Tm_{app} of TA_5182, but for TA_2799, there was a significant increase in the Tm_{app} (Figure S7A and C). Sucrose has a better effect on increasing the stability of TA_2799 as compared to glycerol. In case of TA_5182, however, the increase in the amount of sucrose does not have any effect on the Tm_{app} of the enzyme. A similar trend was observed for the TA_5182_G119N mutant, where the Tm_{app} of the enzyme seems to be unaffected by the addition of sucrose and increased by 5°C on addition of 40% glycerol. A ~5°C shift in the Tm_{app} of the mutant enzyme is observed over its wild-type counterpart (Figure S7).

The single mutant TA_2799_L322F also showed an Tm_{app} increase of 6.1°C over the wild-type enzyme (Figure S7B). Addition of glycerol and sucrose conferred a significant increase in the Tm_{app} of the mutant. The effect of sucrose on the Tm_{app} of the enzyme was more prominent, in the presence of 40% glycerol, the Tm_{app} increased by 9.1°C, while in the presence of 40% sucrose, the Tm_{app} increased by 15.1°C. The increased affinity of the enzyme to its cofactor PLP is the driving cause for the increase in the Tm of the mutant enzyme, as the binding of PLP has been shown to increase the stability of ω-TAs in several studies (26, 27, 35, 36). These results suggest that the mutant enzymes are more stable than the wild type, and the residues F322 (in TA_2799_L322F) and N119 (in TA_5182_G119N) play a key role in the stability of fold type I ω-TAs.

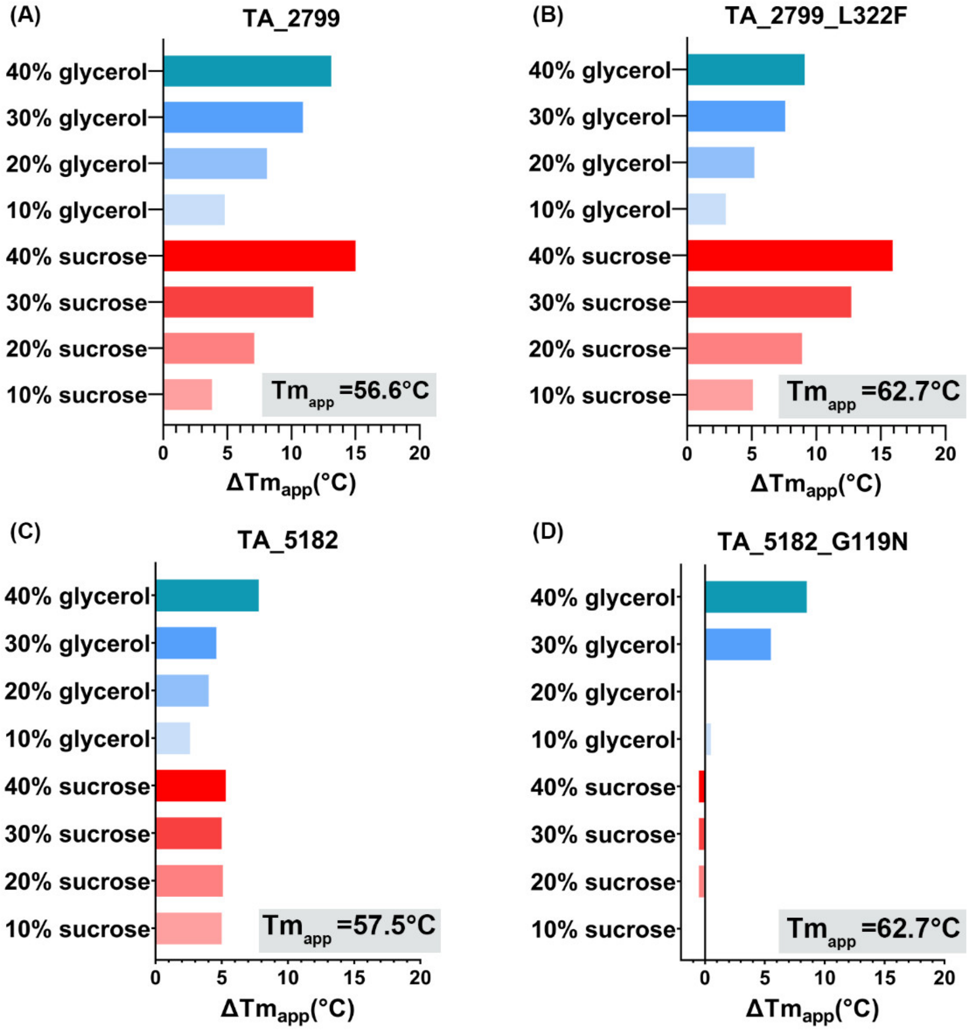

Figure S7: Shift in the Tm_{app} of **(A)** TA_2799, **(B)** TA_2799_L322F, **(C)** TA_5182 and **(D)** TA_5182_G119N in the presence of additives like glycerol and sucrose in varying concentrations. The Tm_{app} of the enzymes in the absence of any additives is denoted in the grey boxes. The Tm_{app} of the enzymes in buffer (50 mM HEPES, pH 8.0) are highlighted in grey boxes. The effect of sucrose on the Tm_{app} of TA_5182 and its mutant is negligible, but both glycerol and sucrose confer increment in the Tm_{app} of TA_2799 and TA_2799_L322F mutant. Each assay was performed in duplicates, and one datapoint was plotted for generation of the graphs.

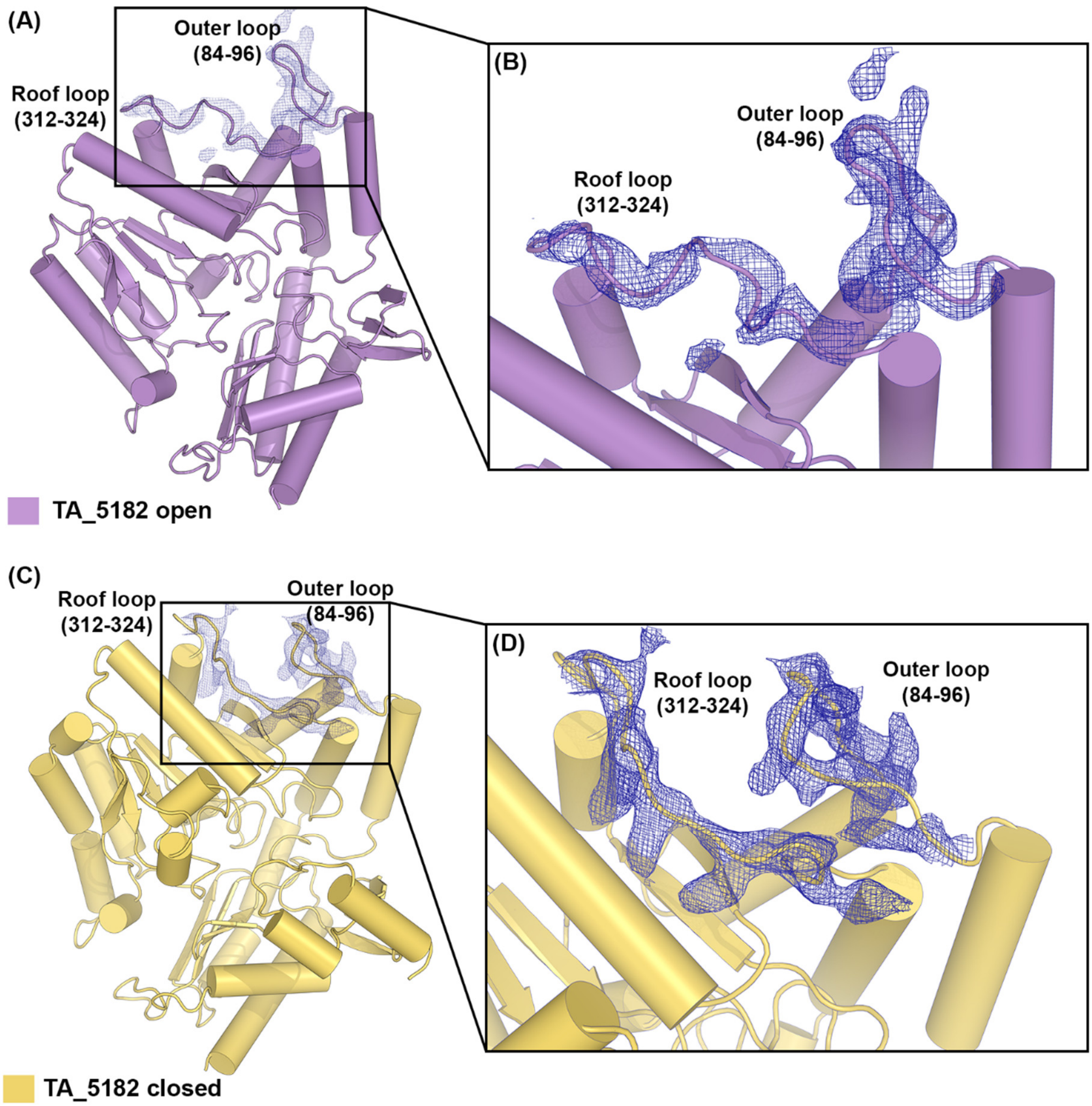

Figure S8: Electron density of the flexible roof loop and outer loop and seen in the crystal structures of **(A-B)** Open state of TA_5182 and **(C-D)** Closed state of TA_5182. For better visualization, a single monomer is shown in cartoon representation and the $2F_o$-F_c electron densities of the loop region at contour level 1.0 σ are shown as blue mesh that represents the electron density around the region. The loops show different conformation (represented in Figure 3A. which mediates the movement of the substrate and cofactor molecule in and out of the enzyme's active site.

Media S1: Overall structure of PLP bound TA_2799 showing different structural features of fold type I ω-TAs: i. Cartoon representation of the monomer with covalently bound PLP at the active site. ii. Cartoon representation of the dimer showing the PLP binding pocket and the flexible loops. iii. Surface representation of the dimer showing the position of the flexible loops and the substrate entry channel of the enzyme.

Media S2: Transition of F321 from the open to closed state in TA_5182 leading to conformational changes in the side chains of residues around the PLP binding pocket of the enzyme. The S122 shows a polar interaction with the carbonyl oxygen of E123. On transition of the enzyme from the open to closed state, the movement of the F321 loop causes rearrangement of the E123 and the helix harbouring S122, and thus S122 is no longer in hydrogen bonding distance with E123, allowing it to coordinate with the phosphate group of the PLP molecule. The PLP molecule has been superposed from the crystal structure of TA_2799. All distances in Å. The ′ symbol denotes the residue from the other subunit. The morph transition has been generated in PyMol using the 'morph' command.

Media S3: Transition of Y323 from the open to closed state in TA_5182 forming hydrogen bond interactions with catalytic K289′ in the closed conformation of the enzyme. In the open state of the enzyme, the polar hydroxyl group of Y323 interacts with T127. In the closed state, the Y323 positions itself underneath the catalytic K289, and interacts with the main chain of K289, thereby stabilizing the catalytic residue. The morph transition has been generated in PyMol using the 'morph' command.

Media S4: Overall transition of the TA_5182 enzyme from open to closed state showing the movement of the loops and the overall change in the structure of the enzyme. The enzyme is relaxed in its open state, while the monomers seem to compact upon each other on transition to the closed state of the enzyme. The outer loop (residues 84-96) and the roof loop (residues 312-324) are coloured solid, with the spheres denoting the Cα atoms of the residues. The lid loop (residues 150-180) could be modelled in this morph due to its absence in the open state of the enzyme. The morph transition has been generated in PyMol using the 'morph' command.

Table S4: Kinetic parameters of the TA_L322F_C324Y

Enzyme	K_m (mM)	V_{max} (U min^{-1})	k_{cat} (min$^{-1)}$	k_{cat}/K_m (mM^{-1} min$^{-1)}$
**TA_2799_L322F_C324Y**	1.188 ± 0.122	0.033 ± 0.001	1.381 ± 0.049	1.162

Table S5: Primers for Site Directed Mutagenesis of the TA enzymes

2799_L322F_f	5′ CCACGGC**TTC**ACCTGCACGGGCCAC 3′
2799_L322F_r	5′ CGTGCAGGT**GAA**GCCGTGGGCGAAT 3′

5182_G119N_f	5′ TTCTTCACC**AAC**TCCGGCTCCGAAGGCAACGAC 3′
5182_G119N_r	5′ GGAGCCGGA**GTT**GGTGAAGAACACATGGGTCAT 3′

2799_L322F_C324Y_f	5′ CCACGGC**TTC**ACC**TAC**ACGGGCCAC 3′
2799_L322F_C324Y_r	5′ CGT**GTA**GGT**GAA**GCCGTGGGCGAAT 3′

2799_I259V_f	5′ GATCGACGAA**GTG**ATCACCGGCTTTGGC 3′
2799_I259V_r	5′ GCCGGTGAT**CAC**TTCGTCGATCACCAC 3′

5182_Y323C_f	5′ GGTTTCACC**TGC**TCCGGCCACCCGGTGG 3′
5182_Y323C_r	5′ GTGGCCGGA**GCA**GGTGAAACCGTGGTTGAA 3′

References:

1. Wilding, M., Scott, C., Newman, J., and Peat, T. S. (2017) Crystal structure of a putrescine aminotransferase from *Pseudomonas* sp. strain AAC. *Acta Crystallogr. F Struct. Biol. Commun.* **73**, 29–35

2. Planchestainer, M., Hegarty, E., Heckmann, C. M., Gourlay, L. J., and Paradisi, F. (2019) Widely applicable background depletion step enables transaminase evolution through solid-phase screening. *Chem. Sci.* **10**, 5952–5958

3. Roda, S., Fernandez-Lopez, L., Benedens, M., Bollinger, A., Thies, S., Schumacher, J., Coscolín, C., Kazemi, M., Santiago, G., Gertzen, C. G. W., Gonzalez-Alfonso, J. L., Plou, F. J., Jaeger, K.-E., Smits, S. H. J., Ferrer, M., and Guallar, V. (2022) A plurizyme with transaminase and hydrolase activity catalyzes cascade reactions. *Angew. Chem. Int. Ed.* **61**, e202207344

4. Humble, M. S., Cassimjee, K. E., Håkansson, M., Kimbung, Y. R., Walse, B., Abedi, V., Federsel, H.-J., Berglund, P., and Logan, D. T. (2012) Crystal structures of the *Chromobacterium violaceum* ω-transaminase reveal major structural rearrangements upon binding of coenzyme PLP. *FEBS J.* **279**, 779–792

5. Crystal structure of a class III aminotransferase from *Silicibacter pomeroyi* [online] https://doi.org/10.2210/pdb3HMU/pdb (Accessed February 17, 2025)

6. Yang, L., Zhang, K., Xu, M., Xie, Y., Meng, X., Wang, H., and Wei, D. (2022) Mechanism-guided computational design of ω-transaminase by reprograming of high-energy-barrier steps. *Angew. Chem. Int. Ed.* **61**, e202212555

7. Sayer, C., Isupov, M. N., Westlake, A., and Littlechild, J. A. (2013) Structural studies of *Pseudomonas* and *Chromobacterium* ω-aminotransferases provide insights into their differing substrate specificity. *Acta Crystallogr. D Biol. Crystallogr.* **69**, 564–576

8. Crystal structure of the W60C mutant of the (S)-selective transaminase from *Chromobacterium violaceum* [online] http://doi.org/10.2210/pdb6snu/pdb (Accessed February 17, 2025)

9. Ruggieri, F., Campillo-Brocal, J. C., Chen, S., Humble, M. S., Walse, B., Logan, D. T., and Berglund, P. (2019) Insight into the dimer dissociation process of the *Chromobacterium violaceum* (*S*)-selective amine transaminase. *Sci. Rep.* **9**, 16946

10. Han, S.-W., Kim, J., Cho, H.-S., and Shin, J.-S. (2017) Active site engineering of ω-transaminase guided by docking orientation analysis and virtual activity screening. *ACS Catal.* **7**, 3752–3762

11. Wilding, M., Peat, T. S., Kalyaanamoorthy, S., Newman, J., Scott, C., and Jermiin, L. S. (2017) Reverse engineering: transaminase biocatalyst development using ancestral sequence reconstruction. *Green Chem.* **19**, 5375–5380

12. Crystal structure of a putative aminotransferase (mll7127) from *mesorhizobium loti* maff303099 at 1.55 A resolution [online] http://doi.org/10.2210/pdb3gju/pdb (Accessed February 17, 2025)

13. Meng, Q., Ramírez-Palacios, C., Capra, N., Hooghwinkel, M. E., Thallmair, S., Rozeboom, H. J., Thunnissen, A.-M. W. H., Wijma, H. J., Marrink, S. J., and Janssen, D. B. (2021) Computational redesign of an ω-transaminase from *Pseudomonas jessenii* for asymmetric synthesis of enantiopure bulky amines. *ACS Catal.* **11**, 10733–10747

14. Palacio, C. M., Rozeboom, H. J., Lanfranchi, E., Meng, Q., Otzen, M., and Janssen, D. B. (2019) Biochemical properties of a *Pseudomonas* aminotransferase involved in caprolactam metabolism. *FEBS J.* **286**, 4086–4102

15. Meng, Q., Capra, N., Palacio, C. M., Lanfranchi, E., Otzen, M., van Schie, L. Z., Rozeboom, H. J., Thunnissen, A.-M. W. H., Wijma, H. J., and Janssen, D. B. (2020) Robust ω-transaminases by computational stabilization of the subunit interface. *ACS Catal.* **10**, 2915–2928

16. Kwon, S., Lee, J. H., Kim, C. M., Jang, H., Yun, H., Jeon, J.-H., So, I., and Park, H. H. (2019) Structural basis of substrate recognition by a novel thermostable (*S*)-enantioselective ω-transaminase from *Thermomicrobium roseum*. *Sci. Rep.* **9**, 6958

17. Crystal Structure of Transaminase from *Shimia marina* [online] https://doi.org/10.2210/pdb8wqj/pdb (Accessed February 17, 2025)

18. Crystal structure of putative aminotransferase (YP_614685.1) from Silicibacter sp. TM1040 at 1.80 A resolution [online] http://doi.org/10.2210/pdb3fcr/pdb (Accessed February 17, 2025)

19. *Bacillus subtilis* BioA in complex with amino donor L-Lys [online] http://doi.org/10.2210/pdb6wnn/pdb (Accessed February 17, 2025)

20. Crystal struture of Aminotransferase PRK07036 from *Rhodobacter sphaeroides* KD131 [online] http://doi.org/10.2210/pdb3i5t/pdb (Accessed February 17, 2025)

21. Guidi, B., Planchestainer, M., Contente, M. L., Laurenzi, T., Eberini, I., Gourlay, L. J., Romano, D., Paradisi, F., and Molinari, F. (2018) Strategic single point mutation yields a solvent- and salt-stable transaminase from *Virgibacillus* sp. in soluble form. *Sci. Rep.* **8**, 16441

22. Dey, S., Lane, J. M., Lee, R. E., Rubin, E. J., and Sacchettini, J. C. (2010) Structural characterization of the *Mycobacterium tuberculosis* biotin biosynthesis enzymes 7,8-diaminopelargonic acid synthase and dethiobiotin synthetase. *Biochemistry*. **49**, 6746–6760

23. Shin, Y.-C., Yun, H., and Park, H. H. (2018) Structural dynamics of the transaminase active site revealed by the crystal structure of a co-factor free omega-transaminase from *Vibrio fluvialis* JS17. *Sci. Rep.* **8**, 11454

24. Crystal structure of omega-transferase from *Vibrio fluvialis* JS17 [online] http://doi.org/10.2210/pdb3nui/pdb (Accessed February 17, 2025)

25. Midelfort, K. S., Kumar, R., Han, S., Karmilowicz, M. J., McConnell, K., Gehlhaar, D. K., Mistry, A., Chang, J. S., Anderson, M., Villalobos, A., Minshull, J., Govindarajan, S., and Wong, J. W. (2013) Redesigning and characterizing the substrate specificity and activity of *Vibrio fluvialis* aminotransferase for the synthesis of imagabalin. *Protein Eng. Des. Sel.* **26**, 25–33

26. Roura Padrosa, D., Alaux, R., Smith, P., Dreveny, I., López-Gallego, F., and Paradisi, F. (2019) Enhancing PLP-binding capacity of class-III ω-transaminase by single residue substitution. *Front. Bioeng. Biotechnol.* **7**, 282

27. Börner, T., Rämisch, S., Reddem, E. R., Bartsch, S., Vogel, A., Thunnissen, A.-M. W. H., Adlercreutz, P., and Grey, C. (2017) Explaining operational instability of amine transaminases: substrate-induced inactivation mechanism and influence of quaternary structure on enzyme–cofactor intermediate stability. *ACS Catal.* **7**, 1259–1269

28. Rausch, C., Lerchner, A., Schiefner, A., and Skerra, A. (2013) Crystal structure of the ω-aminotransferase from *Paracoccus denitrificans* and its phylogenetic relationship with other class III aminotransferases that have biotechnological potential. *Proteins*. **81**, 774–787

29. Wilding, M., Peat, T. S., Newman, J., and Scott, C. (2016) A β-alanine catabolism pathway containing a highly promiscuous ω-transaminase in the 12-aminododecanate-degrading *Pseudomonas* sp. strain AAC. *Appl. Environ. Microbiol.* **82**, 3846–3856

30. Li, M., Wei, Y., Yin, J., Lin, L., Zhou, Y., Hua, G., Cao, P., Ang, E. L., Zhao, H., Yuchi, Z., and Zhang, Y. (2019) Biochemical and structural investigation of taurine:2-oxoglutarate aminotransferase from *Bifidobacterium kashiwanohense*. *Biochem. J.* **476**, 1605–1619

31. Lebedev, A. A., and Isupov, M. N. (2014) Space-group and origin ambiguity in macromolecular structures with pseudo-symmetry and its treatment with the program Zanuda. *Acta Crystallogr. D Biol. Crystallogr.* **70**, 2430–2443

32. Boyko, K. M., Nikolaeva, A. Yu., Bakunova, A. K., Stekhanova, T. N., Rakitina, T. V., Popov, V. O., and Bezsudnova, E. Yu. (2021) Three-dimensional structure of thermostable d-amino acid transaminase from the archaeon *Methanocaldococcus jannaschii* DSM 2661. *Crystallogr. Rep.* **66**, 802–807

33. Artukka, E., Schnell, R., Palmu, K., Rosenqvist, P., Szodorai, E., Niemi, J., Virta, P., Schneider, G., and Metsä-Ketelä, M. (2023) Pseudouridine-modifying enzymes Sapb and Saph control entry into the pseudouridimycin biosynthetic pathway. *ACS Chem. Biol.* **18**, 794–802

34. Galman, J. L., Gahloth, D., Parmeggiani, F., Slabu, I., Leys, D., and Turner, N. J. (2018) Characterization of a putrescine transaminase from *Pseudomonas putida* and its application to the synthesis of benzylamine derivatives. *Front. Bioeng. Biotechnol.* **6**, 205

35. Chen, S., Land, H., Berglund, P., and Humble, M. S. (2016) Stabilization of an amine transaminase for biocatalysis. *J. Mol. Catal. B Enzym*. **124**, 20–28

36. Chen, S., Berglund, P., and Humble, M. S. (2018) The effect of phosphate group binding cup coordination on the stability of the amine transaminase from *Chromobacterium violaceum*. *Mol. Catal.* **446**, 115–123
