# Supplementary figures and images for: Structural insights and rational design of Pseudomonasputida KT2440 omega transaminases for enhanced biotransformation of (R)-PAC to (1R, 2S)-Norephedrine
Source: J Biol Chem. 2025 May 26;301(7):110289. doi: 10.1016/j.jbc.2025.110289 (PMC12226076; doi:10.1016/j.jbc.2025.110289)

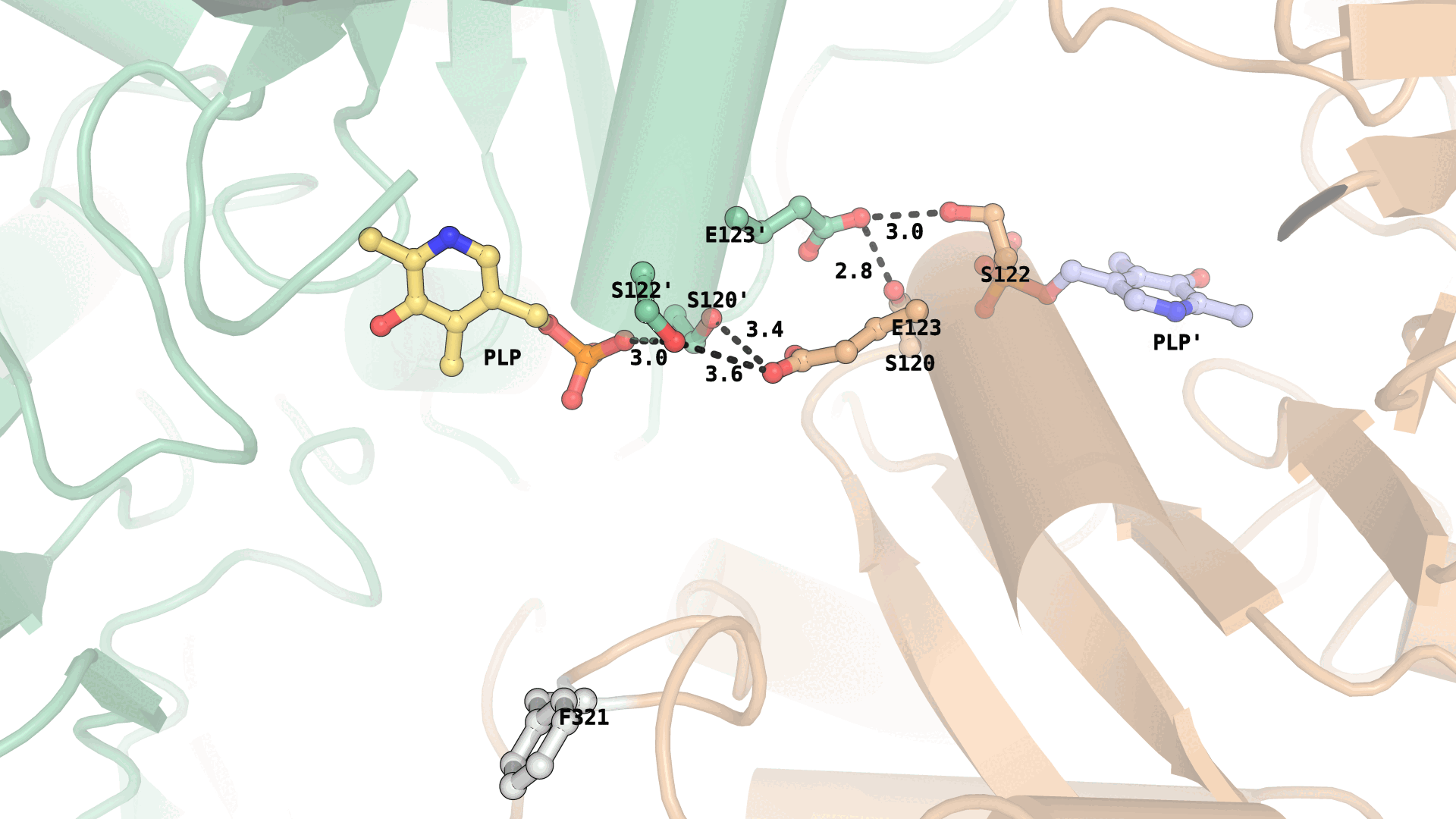

Supplement: Media_S2 [file mmc3.zip › mmc3.gif]

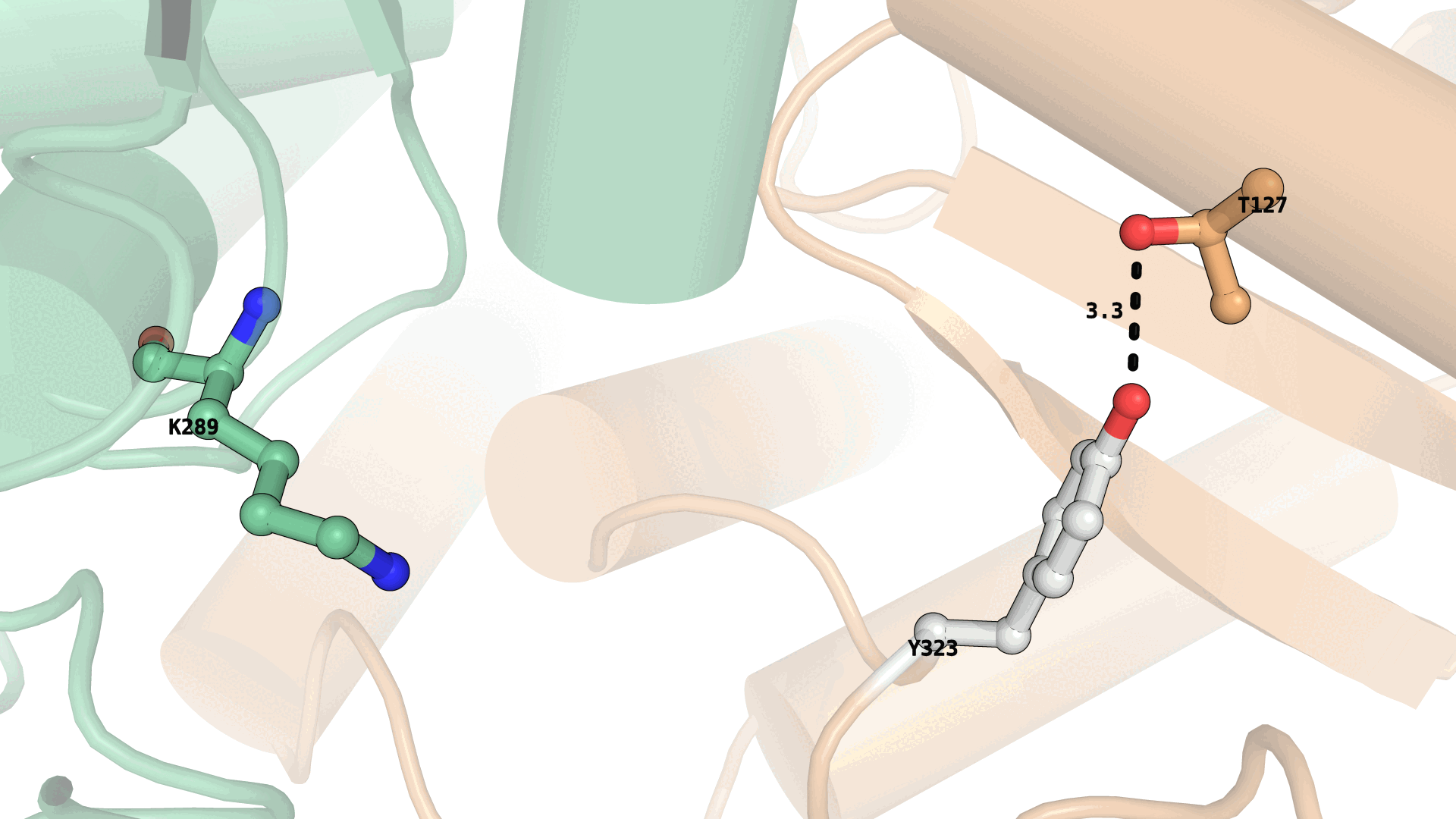

Supplement: Media_S3 [file mmc4.zip › mmc4.gif]
